# Early ground axe technology in Wallacea: The first excavations on Obi Island

**DOI:** 10.1371/journal.pone.0236719

**Published:** 2020-08-19

**Authors:** Ceri Shipton, Sue O’Connor, Shimona Kealy, Mahirta, Indah N. Syarqiyah, Nico Alamsyah, Marlon Ririmasse

**Affiliations:** 1 Centre of Excellence for Australian Biodiversity and Heritage, College of Asia and the Pacific, The Australian National University, Canberra, Australia; 2 Archaeology and Natural History, College of Asia and the Pacific, The Australian National University, Canberra, Australia; 3 Institute of Archaeology, Gordon Square, University College London, London, United Kingdom; 4 Departemen Arkeologi, Universitas Gadjah Mada, Yogyakarta, Indonesia; 5 Pusat Penelitian Arkeologi Nasional, Jakarta, Indonesia; Universita degli Studi di Milano, ITALY

## Abstract

The first excavations on Obi Island, north-east Wallacea, reveal three phases of occupation beginning in the terminal Pleistocene. Ground shell artefacts appear at the end of the terminal Pleistocene, the earliest examples in Wallacea. In the subsequent early Holocene occupation phase, ground stone axe flakes appear, which are again the earliest examples in Wallacea. Ground axes were likely instrumental to subsistence in Obi’s dense tropical forest. From ~8000 BP there was a hiatus lasting several millennia, perhaps because increased precipitation and forest density made the sites inhospitable. The site was reoccupied in the Metal Age, with this third phase including quadrangular ground stone artefacts, as well as pottery and pigs; reflecting Austronesian influences. Greater connectivity at this time is also indicated by an *Oliva* shell bead tradition that occurs in southern Wallacea and an exotic obsidian artefact. The emergence of ground axes on Obi is an independent example of a broader pattern of intensification at the Pleistocene-Holocene transition in Wallacea and New Guinea, evincing human innovation in response to rapid environmental change.

## Introduction

**Fig 1:**
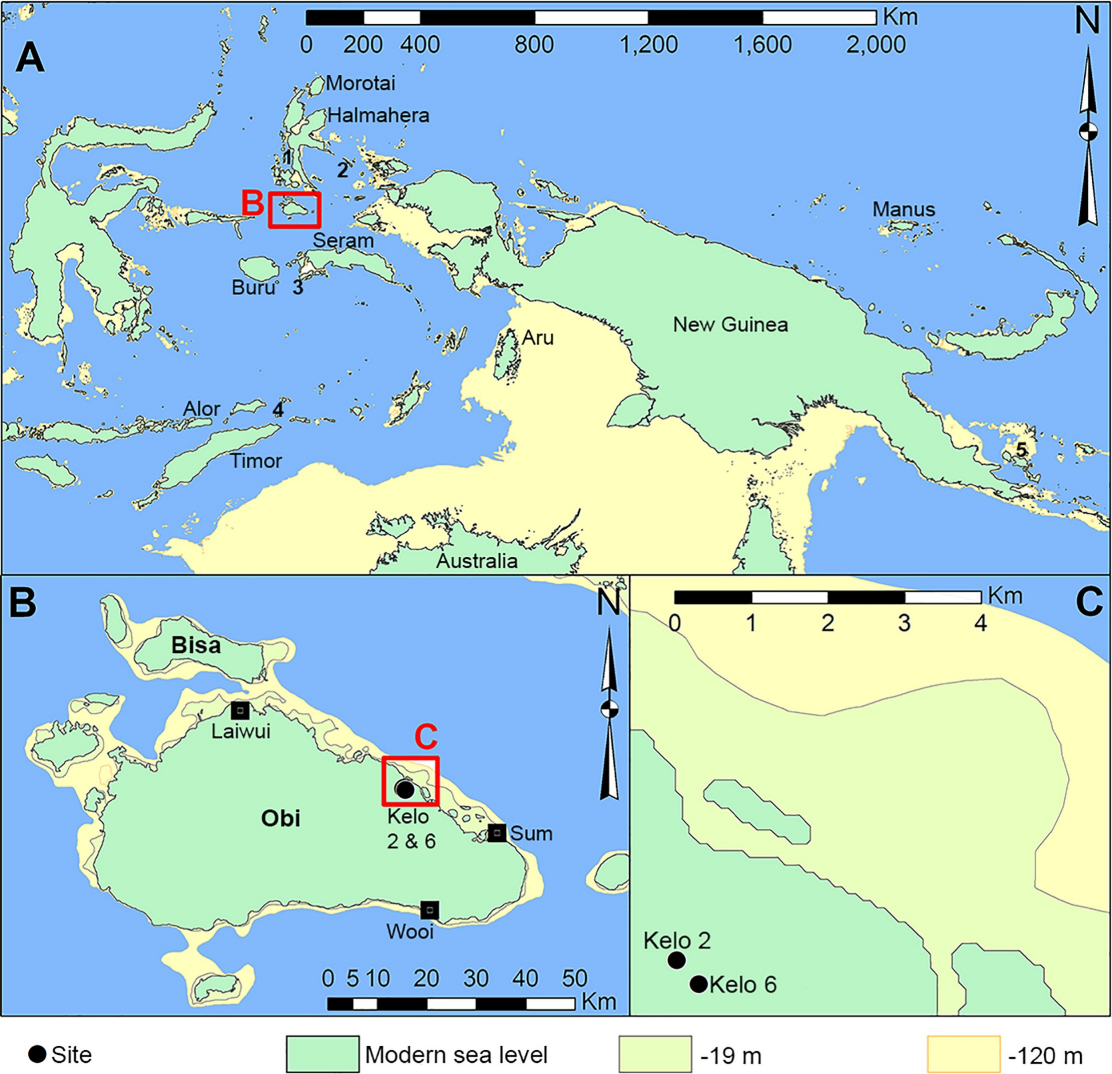
A: Wallacea and New Guinea showing islands mentioned in the text and sea level at -120 m (18 ka). 1 –Kayoa; 2 –Gebe; 3 –Ambon; 4 –Kisar; 5 –Fergusson. B: Obi island showing some villages and extent of island at -120 m sea level. C: The Kelo sites and their distance to the coast at present sea level, -19 m (9 ka), and -120 m (18 ka). Image created by the authors in ESRI’s ArcGIS 10.1. No copyrighted material was used.

Ground axes and adzes are some of the most difficult stone tools to create; requiring both a high level of skill to knap preforms, and a long investment of labour to grind the cutting edge and other parts of the surface [1–5]. They are so clearly artefacts and yet so mysterious as to their process of production to non-knappers, that they were generally thought to be ‘thunderbolts’ in recent folk beliefs, including across Wallacea [6]. Ground axes and adzes are particularly prevalent in, and characteristic of, the Neolithic; being instrumental in the Holocene transition from foraging to farming in many parts of the world [e.g. 7]. But in Australia ground axes have a deep Pleistocene ancestry going back to the initial colonization by *Homo sapiens* over 50,000 years ago [8, 9]. Ground axes were ethnographically known to have been used in raft construction [10], and their occurrence in the early occupation levels at sites in the Arnhem Land and Kimberley regions of northern Australia raises the possibility this technology could have been a Wallacean invention, instrumental in the colonization of Australia. However, current evidence suggests that edge-ground tool technology in Wallacea first emerges in the early Holocene, and in the form of shell adzes rather than stone axes [11–13]. Edge-ground stone tools occur in Borneo to the west and New Guinea to the east of Wallacea from at least the early Holocene and perhaps as early as the Last Glacial Maximum [14, 15]. Quadrangular adzes, associated with the dispersal of the Austronesian Neolithic [16–19], appear in Wallacea during the late Holocene [20–22]. In this paper we report a dated sequence of ground axe/adze technology from new archaeological excavations on the north coast of Obi Island, in the North Maluku archipelago of Wallacea (Fig 1).

**Fig 2:**
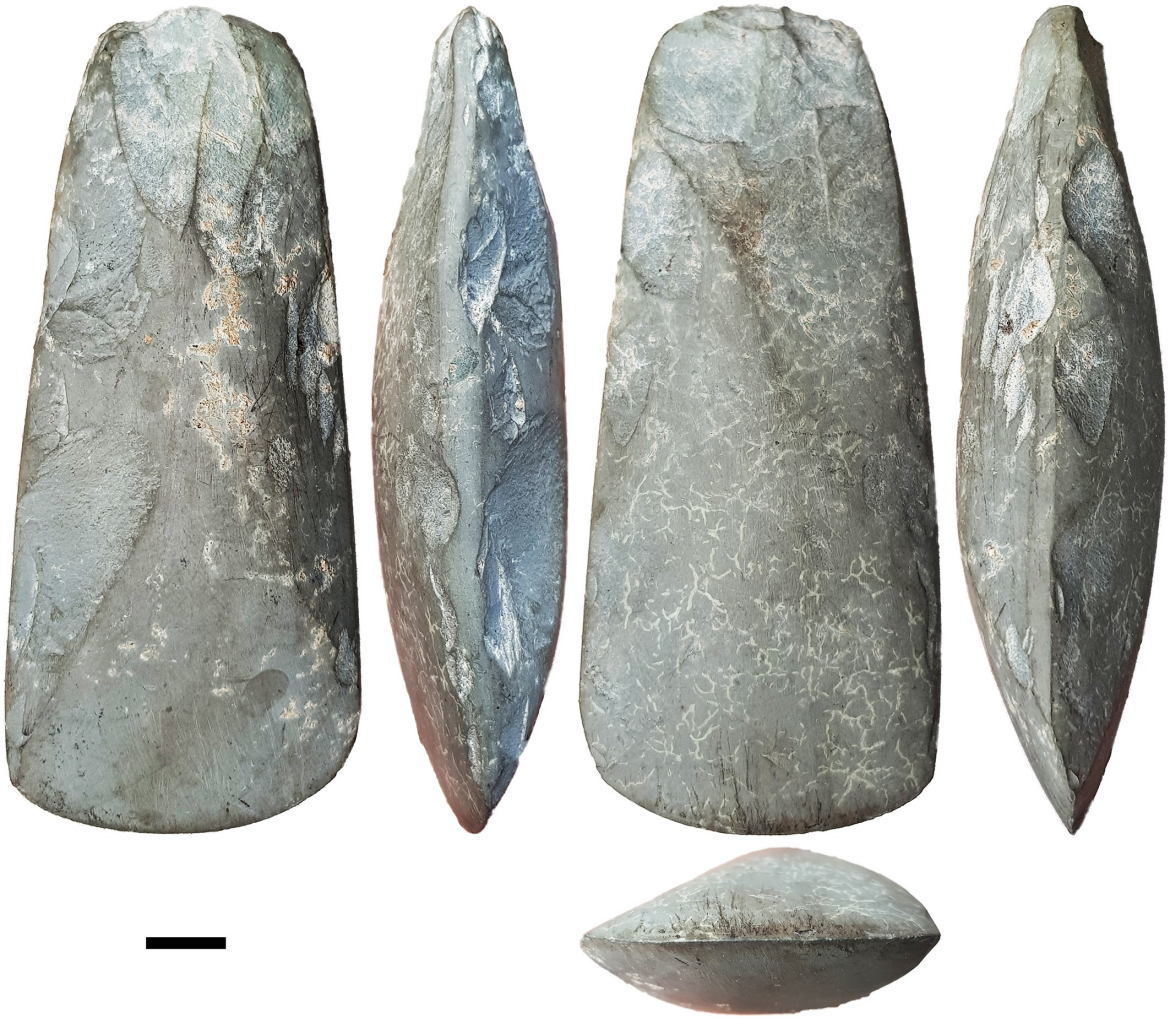
Groundstone axe surface find from near the village of Kelo. Scale bar is 1 cm long.

Human dispersal route modelling indicates Obi Island may have been an important stepping stone between Sunda and Sahul in the Late Pleistocene [23] (Fig 1A). It is a densely forested island, ~2500 km^2^, with a diverse but mostly volcanic geology, and a maximum height of 1611 m amsl. Obi thus presents key promotional conditions for edge-ground axe technology: it is an island that would have required open-water crossings to reach, with stone and shell ground axes/adzes used in the production of water-craft [e.g. 10, 24]; it is densely forested and ground axes/adzes were principally used for heavy-duty wood-working [e.g. 25, 26]; and the two principal materials used for ground axe/adze manufacture are both available on Obi–fine-grained volcanic stone and large tropical shells [e.g. 27]. Survey of the island in 2019 identified several rockshelters in Miocene limestone on the north-central coast near the village of Kelo, with ground axe flakes on the surface. A complete groundstone axe was also recovered from a garden near Kelo (Fig 2). In the following section we describe our excavations at two rockshelters, Kelo 2 and Kelo 6.

## Methods

All necessary permits were obtained for the described study, which complied with all relevant regulations. Permission to conduct this study was granted by the Indonesian Ministry of Research, Technology, and Higher Education (RISTEK) Foreign Research Permit Division (O’Connor 163/FRP/E5/Dit.KI/II/2019). Long-term curation of the excavated material is at the Universitas Gadja Madah, Yogyakarta, Java, Indonesia. All material is stored with labels showing the site code (Kelo) and locality (6/2), as well as the trench name (A), and excavation unit (#).

Excavations at the two Kelo localities was conducted with trowels and proceeded by stratigraphic context (layer) [28] (S1 Fig in S1 File). Layers were differentiated on the basis of sediment colour, texture, and compaction during excavation. Arbitrary ~2.5 cm thick spits following the upper surface of each layer were used as a control within thicker contexts. These excavation units were recorded on *pro forma* sheets detailing depths at five points in each 1x1 m unit, sediment description, sketch plans, and any other observations. Minor bioturbation was noted during excavation, but burrows were emptied and the material discarded. Large pieces of charcoal were collected during excavation for radiocarbon dating. All other excavated material was dry and wet sieved through a 1.5 mm^2^ mesh, dried, and sorted by material class. All excavated sediment and discarded rocks and residue were weighed.

All lithics were examined and assigned a material type and technological class (core, primary flake, secondary flake, tertiary flake, broken flake, retouched flake, flaked piece, heat pop, grinding stone, cobble manuport). Complete flakes had their axial length, medial width, platform thickness, and platform angle measured. Their platform type and dorsal scar pattern was also recorded, and the number of dorsal scars counted. The Scar Density Index (SDI) [29] was estimated by dividing the number of scars by the product of axial length and medial width. For flakes with grinding on both the platform and dorsal surface, the orientation of grinding in relation to the axis of the flake was recorded as being sub-parallel if <20°, or oblique/orthogonal if ≥20°. Cobble manuports were weighed and their maximum and minimum dimensions measured.

Accelerator mass spectrometry radiocarbon measurements for Kelo were conducted at the University of Waikato Radiocarbon Dating Laboratory. Calibrations were made using OxCal v4.3.2 [30] and the IntCal13 curve for charcoal and aragonite seeds or the Marine13 curve for marine shell [33]. ΔR corrections were not applied on the marine shell samples as the local reservoir effect is unknown and elsewhere it only affects the ages by <250 years [29], so it would not alter the attribution of ages to the occupation phases discussed below.

### The Kelo localities and their phasing

**Fig 3:**
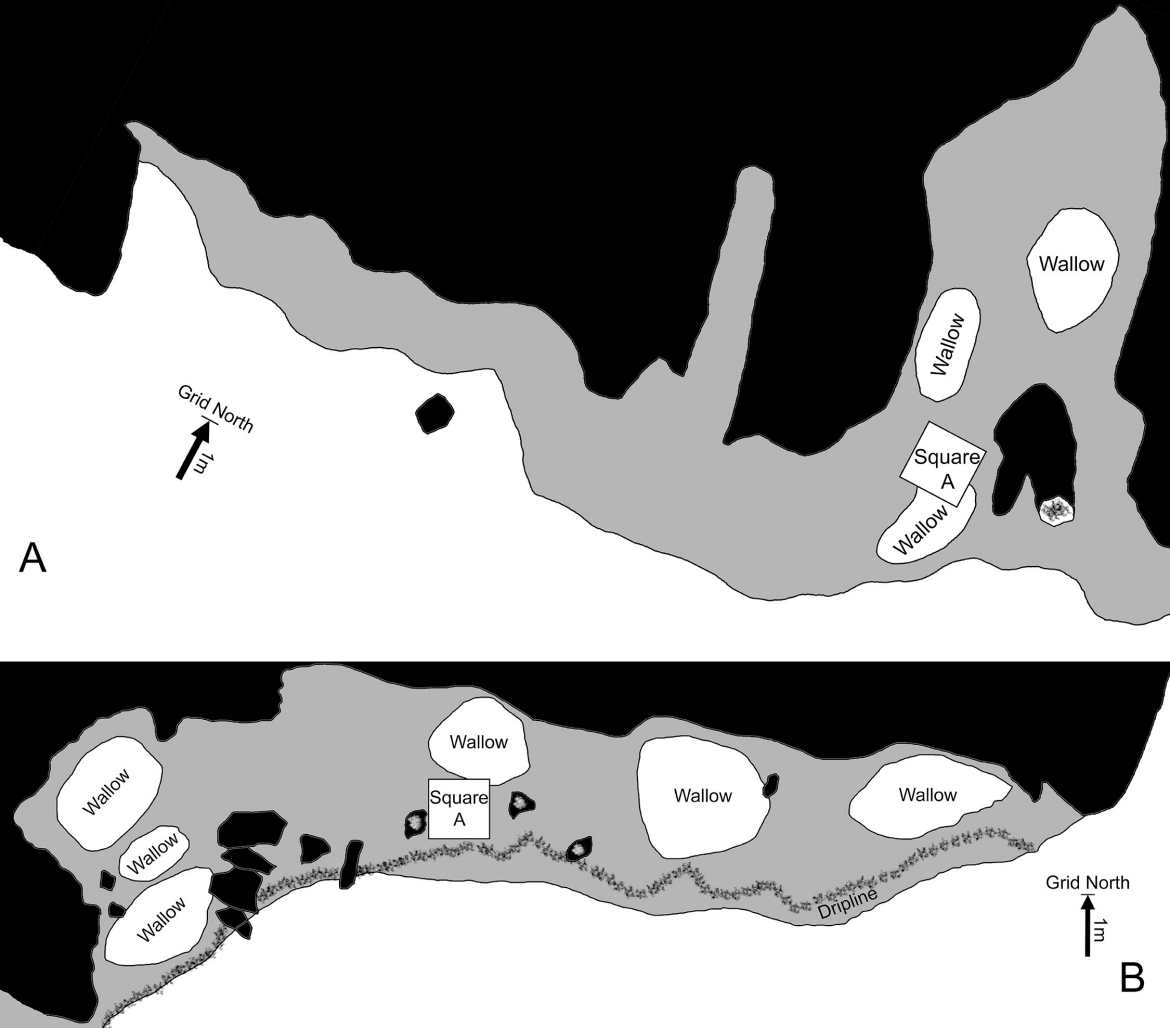
Plan of localities Kelo 2 (A) and part of Kelo 6 (B). Overhanging rock is shown in grey and the shelter wall in black.

**Fig 4:**
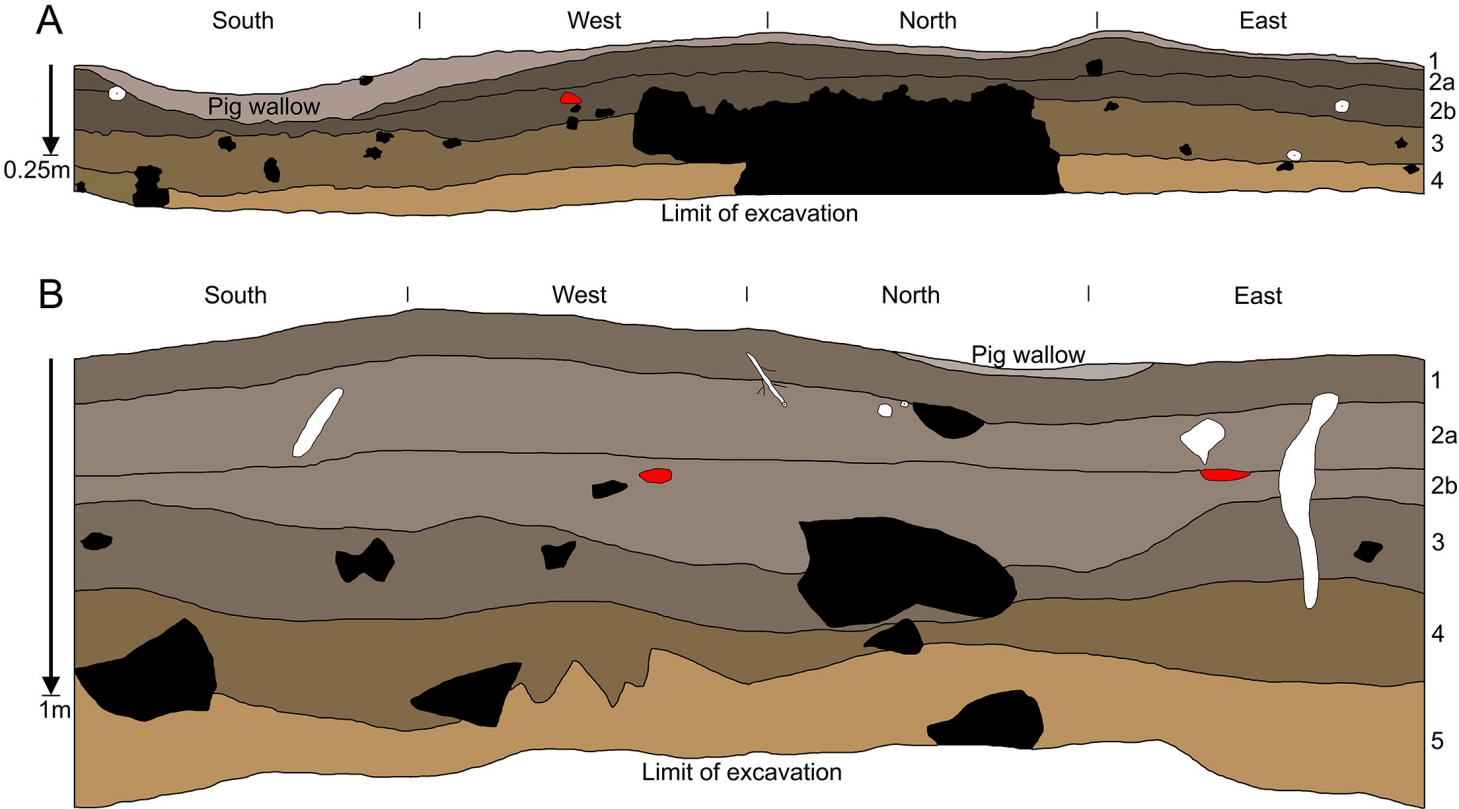
Profile of excavation test pits at Kelo 2 (A) and Kelo 6 (B). Burrows and roots are shown in white, rocks in black, and imported river cobbles in red.

Kelo 2 is a small cave and rockshelter, ~2 km from the coast (Fig 1). Feral pigs have created wallows in the softer areas where more sediment has accumulated (Fig 3). A one metre square test pit was excavated in the entrance to the low-roofed cave, at a spot where a piece of obsidian and an axe flake were found on the surface (Fig 3). The excavation reached archaeologically sterile degrading bedrock at a depth of ~40 cm where it was discontinued. Four layers were differentiated during excavation (Fig 4): Layer 1 (spit 1) was a dry brownish grey (10YR 5/2) loose clayey silt; layer 2 (spits 2–4) was a dry brown (10YR 2/2) firm clayey silt with limestone clasts increasing in frequency with depth; layer 3 (spits 5–8) was a yellowish brown (10YR 3/4) gravelly clay; and layer 4 (spits 9–10) was a brownish yellow (10YR 5/6) moist gravelly clay.

Kelo 6 is a relatively large rockshelter, ~30 m long and ~3 m high, located a few hundred metres further inland from Kelo 2. As with Kelo 2, wallowing by feral pigs has disturbed much of the upper sediment (Fig 3). Several stone artefacts were exposed on the surface, with imported river cobbles in the pig wallows, and knapped lithics, including ground axe flakes, in the drip line. A one metre square test pit, Square A, was excavated approximately in the centre of the main part of the shelter (Fig 3). Sterile sediment with degrading bedrock was reached at a depth of ~1.3 m, at which point the excavation was discontinued. Five layers were differentiated during excavation (Fig 4): Layer 1 (spits 1–4) was a brownish grey (10YR 3/2) loose dry sandy silt; layer 2 (spits 5–12) was a light brownish grey (10YR 4/2) sandy silt grading into clayey silt with depth; layer 3 (spits 13–20) was a moist greyish brown (10YR 3/2) silty clay; layer 4 (spits 21–23) was a moist yellowish brown (10YR 3/4) silty clay; and layer 5 (spits 24–30) was a damp brownish yellow (10YR 5/6) firm gravelly clay. Due to the limestone parent rock, the sediment was alkaline throughout (pH levels ranging from 7.5 to 9.5), which facilitated good preservation of bone and shell. The sub-division of layer 2 into two is based on the finer matrix yet higher number of limestone inclusions in the lower part of the layer (18% vs 10% by weight), with the distinction borne out by the differences in charcoal sample ages between the upper and lower parts of the layer, as well as the lack of pottery and pig bone in the lower part.

**Fig 5:**
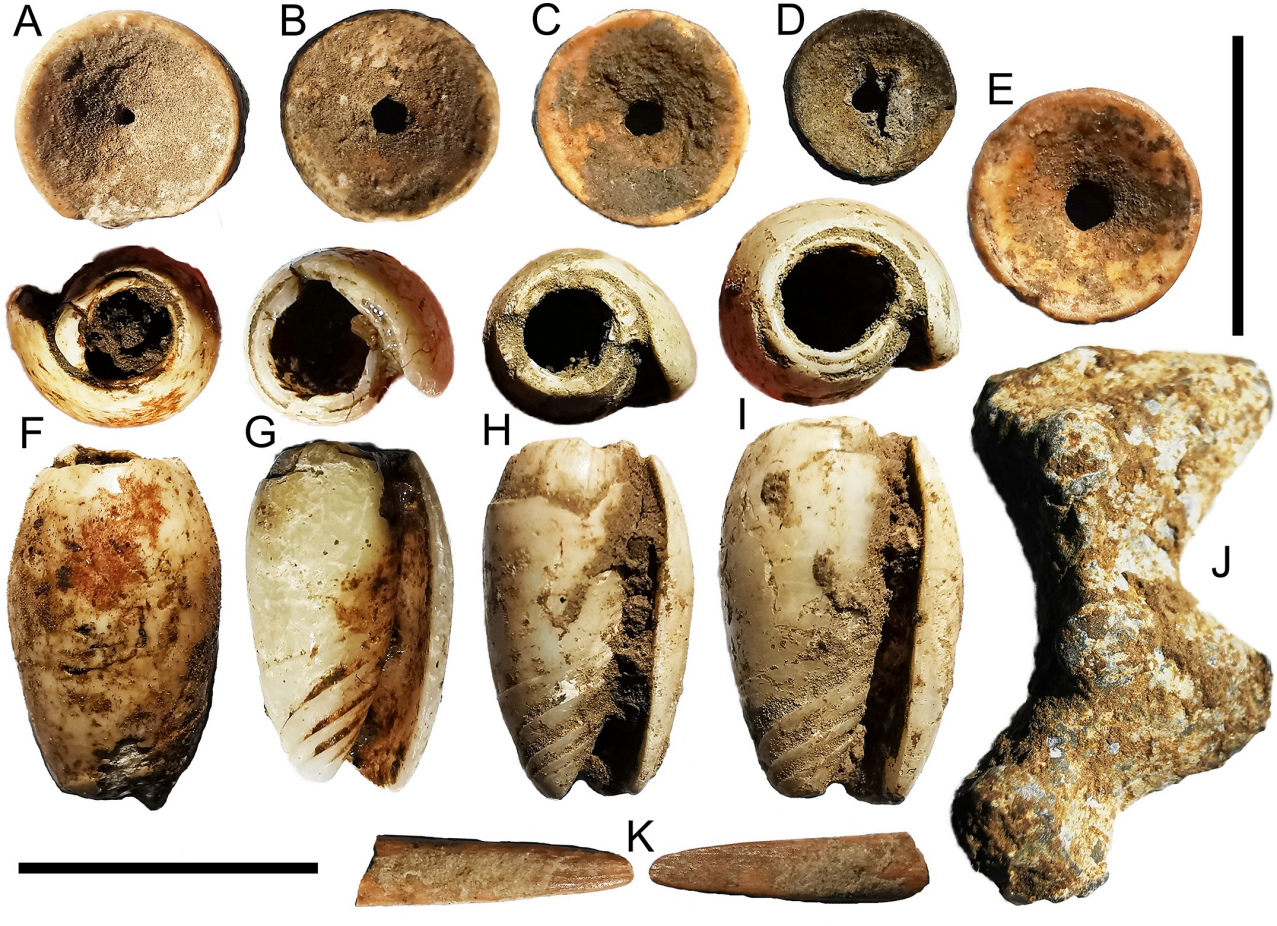
Non-lithic artefacts from Kelo. A, C, and E-K are Metal Age, B and D are early Holocene. A is a possible shark vertebrae bead, though the small size of the hole suggests it could be natural; B-E are shark vertebrae beads; F-I are Oliva shell beads; J is an iron object, possibly a bracelet fragment; K is the tip of bone point. A is from Kelo 6 layer 2a (spit 6), B is from Kelo 6 layer 3 (spit 15), C is from Kelo 2 layer 1 (spit 1), D is Kelo 2 layer 3 (spit 6), E is from Kelo 6 layer 1 (spit 3), F is from Kelo 6 layer 2a (spit 6), G is from Kelo 6 layer 1 (spit 4), H and I are from Kelo 6 layer 2a (spit 5), J is from Kelo 2 layer 2 (spit 2), K is from Kelo 2 layer 2 (spit 4). Note the ochre on Oliva bead F. Scale bars are 1 cm long.

Two radiocarbon dates were obtained on archaeological shell from Kelo 2, from layers 3 (spit 7) and 4 (spit 10); both of which yielded early Holocene ages (Table 1). Layers 1 and 2 (spits 1–4) produced 5.24 g of thin-walled pottery and a metal object weighing 4.41 g (Fig 5J). Volcanic stone flakes are notably more weathered from spit 5 and below. We divide the Kelo 2 sequence into two phases: an upper Metal Age occupation in layers 1–2 (spits 1–4), and a lower early Holocene occupation in layers 3–4 (spits 5–10).

**Table 1:** Radiocarbon dates for Kelo 2 and Kelo 6. Unmodelled and modelled calibrated dates are all shown at 95.4% posterior probability. Modelled dates (including ‘Start’ and ‘End’ dates for each Layer) for Kelo 6 correspond to the Bayesian model performed in OxCal (S1).

LOCALITY	SPIT	LAYER	MATERIAL	LAB CODE, WK:	RADIO-CARBON AGE	ERROR	CALIBRATED RANGE (BP)	MODELLED DATE (BP)
**KELO 2**	A7	3	Marine shell: *Geloina coaxans*	49406	10447	29	11791–11337	N/A
**KELO 2**	A10	4	Marine shell: *Geloina coaxans*	49405	9019	26	9823–9555	N/A
**KELO 6**	End Layer 1							773–511
**KELO 6**	A4	1	Charcoal: unidentified	49422	860	18	795–727	895–524
**KELO 6**	Transition Layer 2a - 1							2142–515
**KELO 6**	A6	2a	Charcoal: unidentified	49421	606	18	651–548	3580–550
**KELO 6**	Start Layer 2a							7635–586
**KELO 6**	End Layer 2b							9068–7374
**KELO 6**	A8	2b	Charcoal: unidentified	49420	7978	22	8993–8727	8999–8755
**KELO 6**	A10	2b	Aragonite seed: *Celtis* sp.	49408	8120	26	9123–9005	9123–8998
**KELO 6**	A10	2b	Charcoal: unidentified	49419	9173	24	10,405–10,247	9329–8298
**KELO 6**	Start Layer 2b							9356–9009
**KELO 6**	End Layer 3							9453–9105
**KELO 6**	A13	3	Charcoal: unidentified	49418	9134	24	10,379–10,230	10,384–10,228
**KELO 6**	A15	3	Charcoal: unidentified	49417	8396	23	9485–9324	9489–9326
**KELO 6**	A16	3	Charcoal: unidentified	49416	10697	34	12,718–12,584	12,745–12,220
**KELO 6**	A17	3	Charcoal: unidentified	49415	9609	25	11,138–10,784	11,165–10,762
**KELO 6**	A19	3	Charcoal: unidentified	49414	8402	23	9488–9331	9496–9322
**KELO 6**	Start Layer 3							13,580–12,448
**KELO 6**	End Layer 4							13,697–12,797
**KELO 6**	A21	4	Charcoal: unidentified	49413	11757	28	13,719–13,464	13,722–13,464
**KELO 6**	Start Layer 4							14,946–13,478
**KELO 6**	End Layer 5							16,609–13,717
**KELO 6**	A24	5	Aragonite seed: *Celtis* sp.	49407	12836	32	15,493–15,143	17,011–15,087
**KELO 6**	A24	5	Marine shell: *Rochia nilotica*	49410	14974	39	17,926–17,589	17,955–17,537
**KELO 6**	A26	5	Marine shell: *Hippopus* sp.	49411	14173	42	16,889–16,405	16,925–16,372
**KELO 6**	A26	5	Marine shell: unidentified	49409	14513	37	17,384–16,985	17,418–16,939
**KELO 6**	A29	5	Marine shell: *Hippopus* sp.	49412	14267	37	16,997–16,566	17,017–16,528
**KELO 6**	Start Layer 5							22,898–17,483

For Kelo 6, 16 radiocarbon dates were obtained on charcoal and *Celtis* sp. seeds for the upper layers, and marine shell and *Celtis* sp. seeds for the lower layers (Table 1). Two dates from layers 1 and 2a (spits 4 and 6) are in the early last millennium, eight dates from layers 2b and 3 (spits 8–19) are early Holocene (8700–12,700 BP), and six dates from layers 4 and 5 (spits 21–29) are terminal Pleistocene (13,400–17,900 BP). A total of 10.22 g of thin-walled pottery was recovered from layers 1 and 2a (spits 2–7) of Kelo 6. We divide the Kelo 6 sequence into three phases: a Metal Age occupation in layers 1-2a (spits 1–7), an early Holocene occupation in layers 2b-3 (spits 8–20), and a terminal Pleistocene occupation in layers 4–5 (spits 21–30).

A multiphase Bayesian model was constructed for Kelo 6 in OxCal v4.3.2 [30] to reduce uncertainty in the age estimates, predict the timing of transitions between the different identified stratigraphic layers, and to test the presence of a hiatus in the record. Each layer identified in the stratigraphy (Fig 4, Table 1) was modelled as a *Phase* and ordered based on its stratigraphic position. Double boundaries were inserted between each layer to account for discontinuous sedimentation rates and possible hiatuses in the record. One exception was between Layer 2a and Layer 1 where only a single boundary was placed, based on the continuity of archaeological materials and calibrated dates which group these two layers in the Metal Age period (see above). The General t-type Outlier Model [31] and a prior outlier probability of 5% was applied to the overall model and each individual date, respectively. This model is the one most commonly used for archaeological dates in determining the probabilities of outliers and the scale of offset applied to the data within the model [31, 32].

The Bayesian model found good support for continuity in the Kelo 6 record between layers 5 and 2b (S2 Fig in S1 File), with a modelled estimate of 22,898–17,483 BP for initial deposition of layer 5. The model also recovered a discontinuity in the record between layers 2b and 2a, which further analysis revealed to represent a statistically significant hiatus in the chronological record of Kelo 6, at 95.4% probability (S1 Table and S3 Fig in S1 File). The absence of a sharp erosive boundary between layers 2a and 2b as well as the lack of intervening dates from Kelo 2, supports the notion that this was both an occupational and depositional hiatus.

### Occupation intensity, subsistence, and non-lithic artefacts

**Fig 6:**
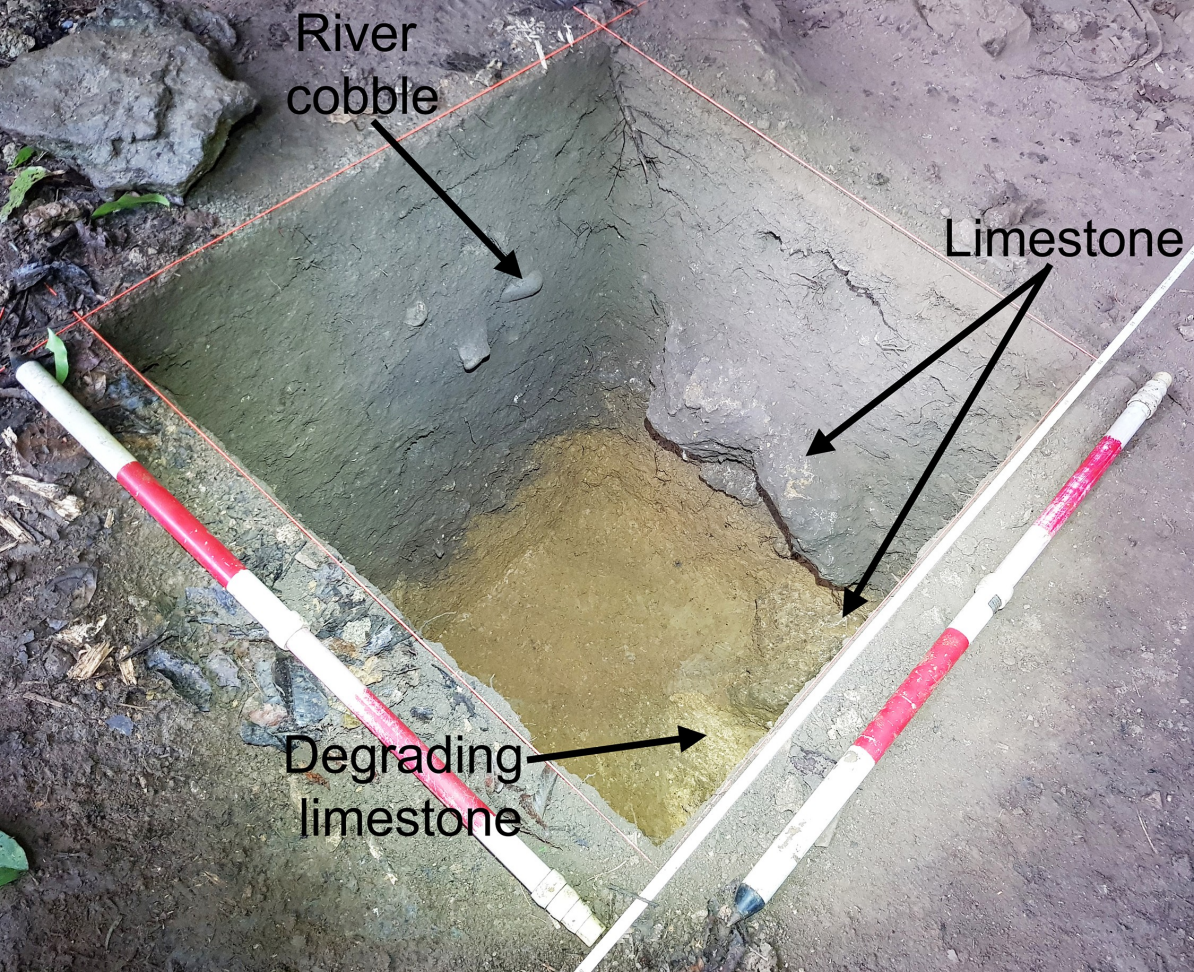
Kelo 6 square a after excavation looking north west. Note the yellow limestone and degrading limestone at the base of the trench and the rounded river cobble manuport in the section. Scales are 1 m long.

The lowermost layers at Kelo 6 and Kelo 2 are yellow in colour because the clay they are composed of derives largely from weathering of the limestone bedrock, which is also yellow when it degrades (Fig 6). With more anthropogenic input the sediment coarsens to silt, becoming first brown then grey. The terminal Pleistocene layers 5–4 in Kelo 6 have a higher ratio of rock to sediment, compared with the early Holocene layers 3-2a and the Metal Age layers 2b-1 (Table 2). This indicates a slower rate of sediment accumulation relative to roof spalling in the terminal Pleistocene, also likely due to less anthropogenic input. Aragonite *Celtis* sp. (hackberry) seeds occur at both sites and are often burnt, suggesting they may have been introduced to the sites by humans. The density of shell, bone, and *Celtis* sp. seeds increases abruptly from the terminal Pleistocene to the early Holocene, further indicating an increase in occupation intensity (Table 2).

**Table 2:** Rock to sediment ratio and shell, bone, and lithic density for Kelo 6. Shell, bone, and Celtis sp. seed density in grams per kilogram of sediment, lithic density in artefacts per kilogram of sediment.

	Rock to sediment ratio	Shell density	Bone density	Lithic density	*Celtis* sp. seed density
**Metal Age**	0.203	10.96	3.67	0.796	0.65
**Early Holocene**	0.234	13.86	2.74	0.236	0.474
**Terminal Pleistocene**	0.402	0.89	0.19	0.264	0.002

Preliminary assessment of the 3188 g of bone excavated from Kelo 6 and Kelo 2 was able to assign 43% to taxa by weight. The dominant fauna is the cuscus *Phalanger rothschildii*, with over 80% of the identified bone in all phases at Kelo 6 and Kelo 2 belonging to this species. Other vertebrates include a large and small murid, bats, Squamata (lizards and snakes), turtles, and fish. In the Metal Age of Kelo 6, 2.8% of identified taxa by weight (14.71 g) are pigs. Of the 13,026 g of mollusc shell from all phases at Kelo 6 and Kelo 2, 97% is marine, with small proportions of freshwater and land snail. Barnacles, crab, and urchin are also represented in low numbers.

In addition to the small quantities of thin-walled pottery mentioned above, non-lithic artefacts include shark vertebrae beads from the early Holocene and Metal Age layers at both Kelo 2 and Kelo 6 (Fig 5A–5E). A bone point, a metal bracelet fragment, and four *Oliva* shell beads were recovered from the Metal Age layers (Fig 5F–5I). One of the *Oliva* beads has ochre on it (Fig 5F), while a single small ochre crayon was found in the early Holocene (layer 2b, spit 9) at Kelo 6.

### Lithics overview

A total of 585 stone artefacts were recorded: 52 from the Kelo 2 excavation and 3 from the surface; with 521 from the Kelo 6 excavation and 9 from the surface. For the following analyses the surface artefacts are included with the Metal Age phase. Despite the low occupation intensity in the terminal Pleistocene, lithic artefact density is marginally higher than in the early Holocene, while across the early Holocene to Metal Age boundary there is a marked increase in lithic density (Table 2). This latter pattern is mirrored at Kelo 2, where lithic density increases sharply from 0.03 artefacts per kilogram of sediment in the early Holocene, to 0.451 artefacts per kilogram of sediment in the Metal Age.

**Fig 7:**
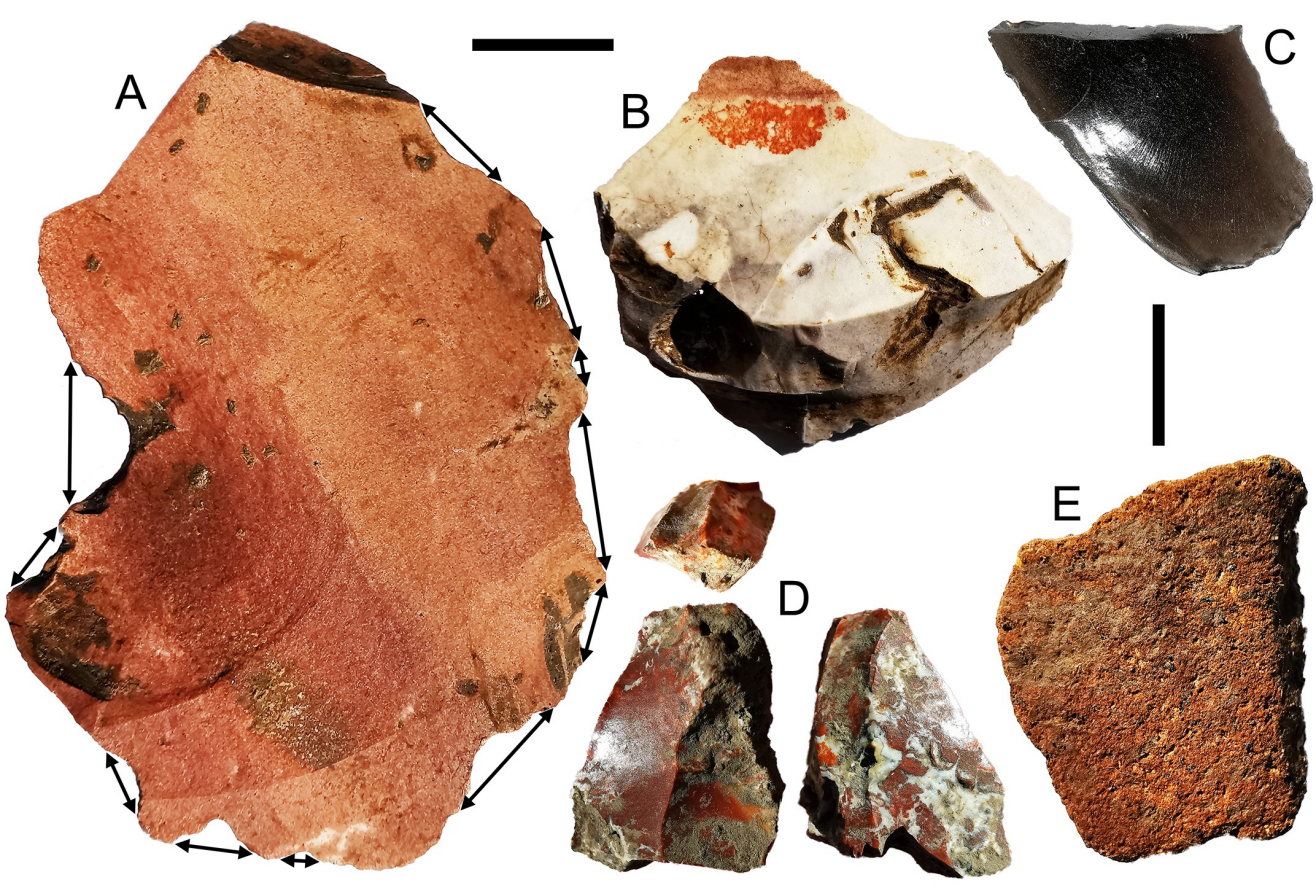
Stone artefacts from Kelo 2 and Kelo 6. A is a chert multiple notched flake from Kelo 6 layer 3 (spit 18, early Holocene); B is a chert nosed-scraper with red residues from Kelo 6 layer 3 (spit 19, early Holocene); C is an obsidian flake from the surface of Kelo 2 (Metal Age). D is a chert bipolar core with invasive gloss on both main surfaces from Kelo 6 layer 2a (spit 5, Metal Age), and E is an igneous grinding stone fragment from Kelo 6 layer 3 (spit 14, early Holocene). Scale bars are 1 cm long.

Eight rounded river cobble manuports occur in the early Holocene and Metal Age phases, identified on the surface and in the excavation (Figs 4 and 6). These are of soft igneous rock, ranging in weight from 0.275 to 0.655 kg, in maximum dimension from 8 to 10 cm, and minimum dimension from 5 to 6 cm, with all fitting comfortably in one hand. They may well have been used as mullers in conjunction with the grinding stones recovered, but the softness of the rock and their origin as river cobbles precludes identification of grinding striations. One of the cobbles is of a harder rock and has two divots on one surface that may have resulted from it being used as a hammerstone. A possible complete grindstone occurred in layer 1 (spit 1) of Kelo 6 (Metal Age), while four grindstone fragments were found in Kelo 2 layer 2 (spit 3) (Metal Age) and Kelo 6 layers 2a (Metal Age), 2b (early Holocene), and 3 (early Holocene) (Fig 7E).

Four materials were used for knapped artefacts: chert, quartz, shell, and a fine-grained, probably basaltic, igneous rock. In addition, there were three coarse metamorphic artefacts of the same type as the grindstones from Kelo 6, and a single obsidian artefact from the surface of Kelo 2 (Fig 7C). No obsidian sources are known on Obi, so this latter artefact is presumed to be exotic. Chert is the dominant material by artefact count in each phase, particularly in the terminal Pleistocene (Table 3). There are small proportions of quartz throughout, but more in the Metal Age (Table 3). Several shell artefacts occur in the terminal Pleistocene but relatively few in the early Holocene and Metal Age, while there are no fine-grained igneous artefacts in the terminal Pleistocene but substantial proportions in the early Holocene and Metal Age (Table 3).

**Table 3:** Breakdown of knapped materials by phase from Kelo 2 and Kelo 6. Other includes coarse-grained metamorphic stone and obsidian.

	Chert	Quartz	Shell	Igneous	Other	Total
**Metal Age**	221 (63%)	15 (4%)	7 (2%)	105 (30%)	1 (1%)	349 (100%)
**Early Holocene**	78 (60%)	1 (1%)	4 (3%)	45 (35%)	2 (1%)	130 (100%)
**Terminal Pleistocene**	79 (84%)	2 (2%)	12 (13%)	0	1 (1%)	94 (100%)
**Total**	378	18	23	150	4	573

Of the 18 quartz artefacts, 5 are cores (28%), 1 is retouched (6%), and only 2 of the complete flakes have cortex (20%). By contrast, of the 378 chert artefacts, only 4 are cores (1%), 7 are retouched (2%), while 46% (total N = 195) of the complete flakes have cortex. This indicates that before being deposited at the site, quartz artefacts tended to be curated for longer than chert with relatively more multiple reduction phase artefacts (cores and retouched flakes) and less on-site knapping creating cortical and unretouched flakes. A Mann-Whitney U test confirmed that scar densities are significantly higher for complete quartz flakes (median = 35.9) than for chert (median = 13.8) (N = 194, U = 427, p = .004), indicating longer reduction sequences for quartz. While chert is locally available near Kelo, the quartz at the site was likely imported from further afield. On complete chert flakes, scar density is significantly greater in the Metal Age (mean = 25.4) than the early Holocene (mean = 16.9) (unequal variances t-test: df = 134.1, t = 2.57, p = .011), but not between the early Holocene and the terminal Pleistocene (mean = 12.7) (equal variances t-test: df = 67, t = 1.079, p = .284).

Nine chert and quartz cores are present in the assemblage, with six being bipolar (Fig 7D), two multi-platform, one discoidal, and one core-on-flake. The presence of crushed (21%, N = 204) and dihedral platforms (7%, N = 204), is likely from bipolar and discoidal knapping respectively, while four Janus (2%, N = 193) dorsal scar patterns confirm the use of core-on-flakes. Chert and quartz flake length is consistent across the three phases (one-way ANOVA: df = 217, F = 0.249, p = .78, mean = 14.99 mm), as is flake elongation (one-way ANOVA: df = 202, F = 1.932, p = .148, mean = 1.41), platform angle (one-way ANOVA: df = 160, F = 1.331, p = .267, mean = 71°), and the proportion of parallel versus orthogonal dorsal scar patterns (N = 180; χ^2^ = 0.672, p = .715). Heat pops (pot lids) occur on twelve of the complete chert flakes (6%, N = 195), with six artefacts themselves classified as heat pops.

Eight retouched artefacts occur in the assemblage, two of which are particularly noteworthy. One is a large flake with 11 individual notches around its perimeter ranging in width from 5.75 to 11.95 mm (Fig 7A). The second is a large flake with small retouch scars on the dorsal around a protrusion, and, on the ventral side of this, two different types of red residue adjacent to each other with an approximately straight border between them (Fig 7B). A bipolar core from the Metal Age was apparently used as a tool with glossy residue left on two opposing surfaces (Fig 7D). The functions of these artefacts are unknown but their use in different types of plant working seems likely: Spokeshaving woody plants creates notches [33]; some trees on Obi have red sap that might explain one of the red residues; and working silica rich plants can leave an invasive gloss on both surfaces of a lithic [34–36].

### Axe flakes

**Fig 8:**
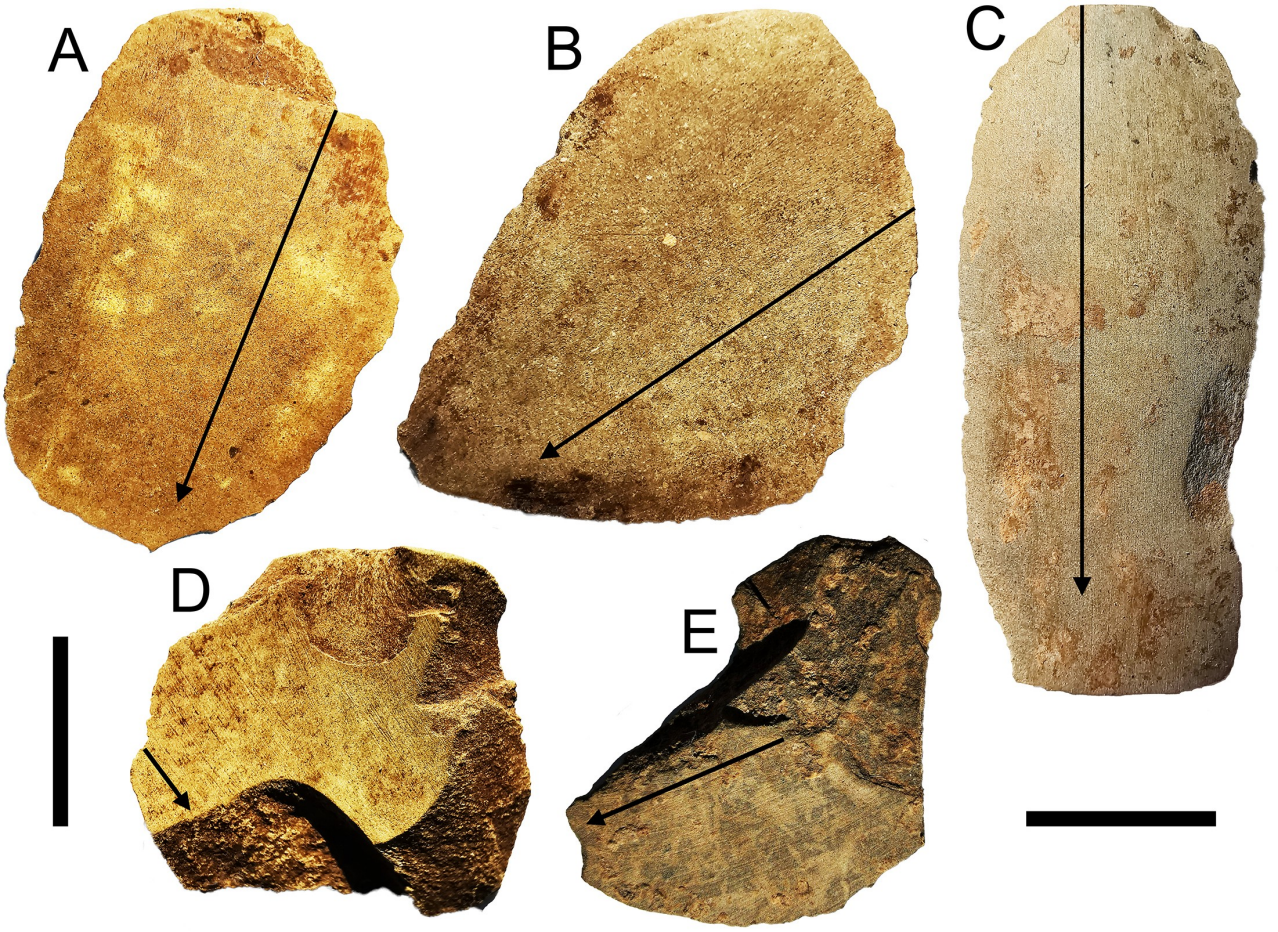
Axe flakes from Kelo 6 with pronounced grinding striations. The arrows denote the dominant orientation of the striations. A and C are from layer 2a (spit 7, Metal Age), B is from layer 1 (spit 4, Metal Age), D is from layer 2b (spit 8, early Holocene), E is from layer 2b (spit 10, early Holocene). For flake C the grinding occurred after the flake scars were struck, while for flakes D and E the grinding is truncated by flaking, presumably to rejuvenate the axe. Flakes A-D also have ground platforms. Scale bars are 1 cm long.

**Fig 9:**
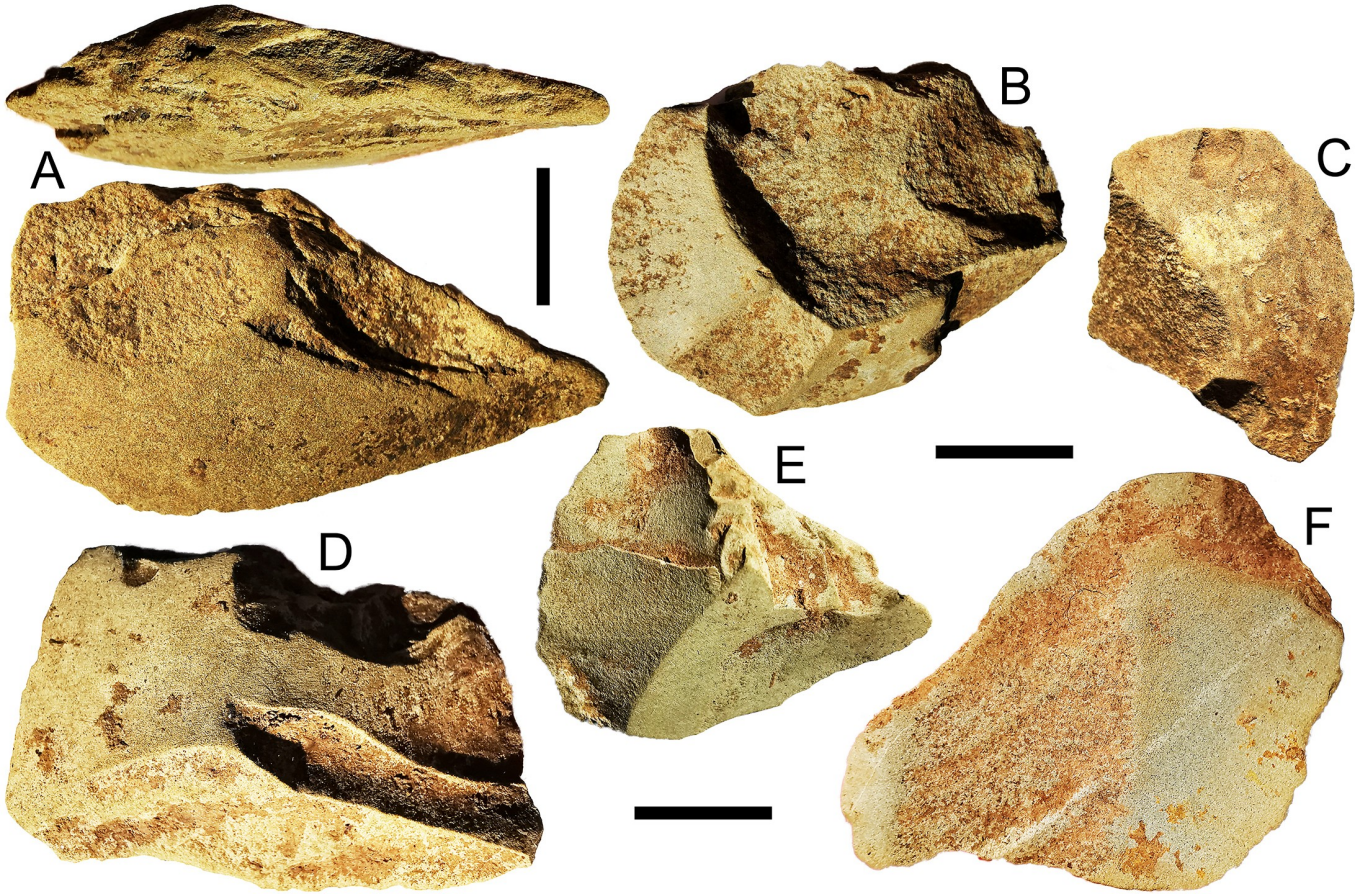
Early Holocene ground axe flakes from Kelo 6. A and C are from layer 3 (spits 20 and 13); B, D, E, and F are from layer 2b (spits 9, 13, 12, and 8). Flakes B-E have flake scars struck from the opposite direction of the flake itself. For flakes B, D, and F there are scars which were struck before and after grinding, while for flakes C and E the scars truncate the grinding. Scale bars are 1 cm long.

Of the 150 igneous flakes in the Kelo assemblage, 76 (51%) have grinding on the dorsal surface (Fig 8), or platform, or both. The igneous material of these flakes is finer than that of the grinding stones, so they are from a distinct tool type. Grinding on two adjacent edges in 33 cases indicates they were struck from groundstone axes. The deepest igneous flakes occur at the beginning of the early Holocene layer 3 in Kelo 6 (spit 20) and include a ground piece (Fig 9A) (S2 Table in S1 File), indicating the link between this material and axe production from the outset.

When considering the 114 complete igneous flakes, only 4% (4) have cortex on them, as opposed to 45% (N = 204) of chert and quartz complete flakes. Some flake scars on igneous lithics are truncated by grinding on the dorsal surface, but in many cases the scars truncate the grinding indicating they were struck to rejuvenate the edge rather than in initial shaping (Fig 8). There is thus an emphasis on resharpening of axes made elsewhere in this igneous flake assemblage.

For igneous flakes with grinding on both the platform and dorsal surface, edge angles are significantly greater where grinding striations on the dorsal are orthogonal to the axis of flaking (median = 80°) versus those with edge angles sub-parallel to the axis of flaking (median = 62°) (Mann-Whitney U test: N = 33, U = 43.5, p = 0.001). Since grinding slicks are typically oriented along the long axis of the tool, this difference in edge angles is likely because sub-parallel striation flakes were struck from the cutting edge (N = 17), whereas orthogonal striation flakes were struck from the sides (N = 16) (S4 Fig in S1 File).

**Fig 10:**
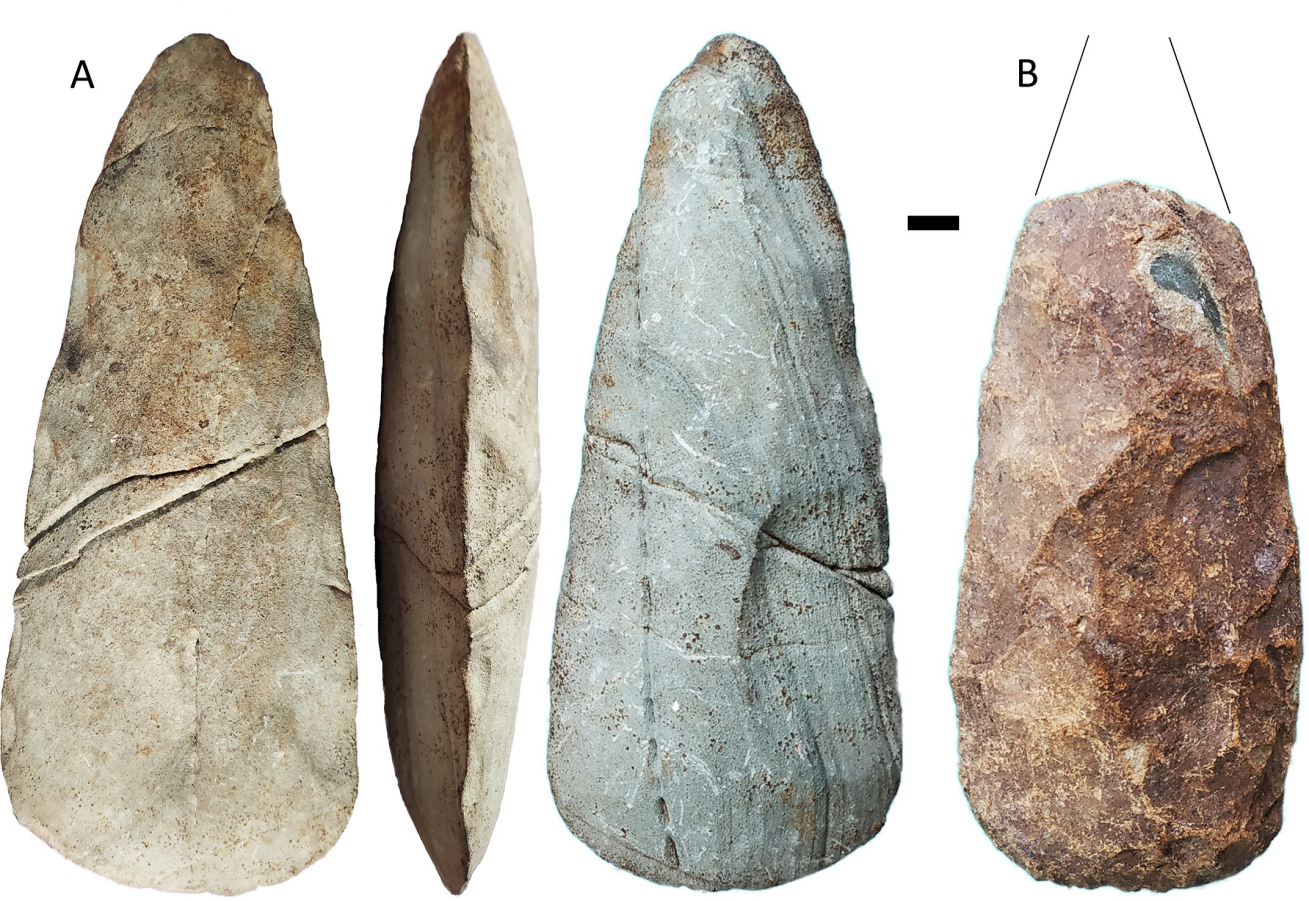
Two bifacial axe surface finds from near the village of Wooi in south-eastern Obi. A appears to be made on sedimentary rock, while B is volcanic with some damage to the surface and the proximal portion broken off. Scale bar is 1 cm long.

On the igneous flakes without ground platforms there is a high proportion of dihedral platforms (18%, N = 74) compared with chert and quartz flakes (7%, N = 205) (χ^2^ = 7.172, p = .007). This accords with the working of bifacial axes such as that shown in Fig 2, where the most prominent part of the edge which serves as a platform is often the meeting point of two scars on the underlying surface. The rarity of cortical platforms, with only a single example known, shows that the axes had adjacent worked surfaces, in other words they had at least two knapped faces. On unground igneous flakes, 28% (N = 54) exhibited scars emanating from the opposite direction to that of the flake itself, with some ground igneous flakes also having these distally struck scars (Fig 9B–9E). These scar patterns indicate bidirectional flaking across surfaces, which allows for shaping of large stone tools such as axes (S4 Fig in S1 File). In addition to the Kelo axe shown in Fig 2, two complete bifacially shaped stone axes were observed as surface finds collected near the village of Wooi, 25 km away in south-eastern Obi (Fig 10).

**Fig 11:**
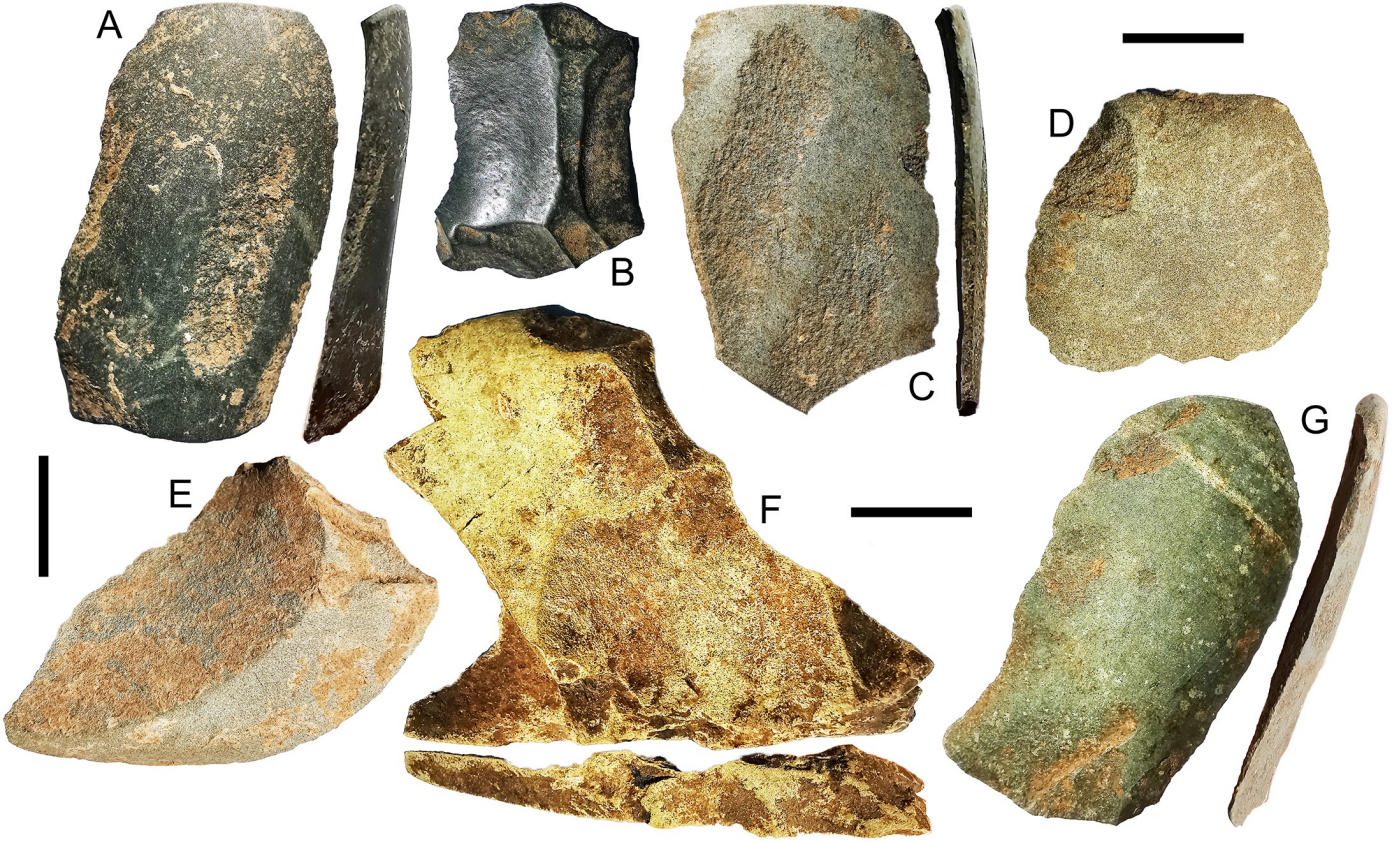
Metal Age axe flakes with both ground platforms and dorsal surfaces from Kelo 6 and Kelo 2. A and C are from the surface of Kelo 6, B is from Kelo 6 layer 1 (spit 2), D is from Kelo 6 layer 2a (spit 7), E and F are from Kelo 6 layer 1 (spit 4), G is from the surface of Kelo 2. For flakes A, C, and G the grinding is parallel to the axis of flaking and they have shallow edge angles, indicating they were likely struck from the cutting edge. For flakes B, and D- F the grinding is oblique or orthogonal to the axis of flaking indicating they were likely struck from the side of the piece. Note that F is an overshot flake preserving a platform on the opposite end to which it was struck. E also preserves the opposite site of the adze, but in this case the profile is rounded rather than squared. Note the polishing on flake B. For flakes A and F the grinding is truncated by flake scars while for flakes A-C, and G the grinding obscures old scars. Scale bars are 1 cm long.

A key difference between axe production in the early Holocene and Metal Age appears to be the appearance of quadrangular forms in the latter. Platform angles on igneous flakes with at least one ground surface are significantly higher in the Metal Age (median = 74°) than the early Holocene (median = 63°) (Mann-Whitney U test: N = 62, U = 270, p = .017). A large redirecting flake struck from a single scar platform from the surface of Kelo 6 preserves a bidirectional platform on its lateral edge (S5 Fig in S1 File), indicating that it had at least three orthogonal knapped surfaces. An overshot flake from Kelo 6 layer 1 (spit 4) was struck from a quadrangular piece as indicated by the angles between the dorsal surface and both the ground platform and the platform preserved on the distal end being ≥80° (Fig 11F). The main dorsal surface was bidirectionally flaked as is necessary for quadrangular tool production [5], with the length of this overshot flake, 39.35 mm, corresponding to the width of the tool it was struck from. In the early Holocene sample, there was only one igneous flake with a complex platform formed from three or more scars, but these constitute 29% (total N = 52) of Metal Age unground platforms on igneous flakes. This is perhaps due to squaring the edges in quadrangular adze knapping, whereby multiple short flakes are removed along a continuous straight edge prior to rotating the piece and flaking at right angles using those small scars as a platform [5]. One of the Metal Age igneous flakes is polished, an attribute not seen in the early Holocene (Fig 11B).

Bifacial, rounded axes were still in use in the Metal Age, as indicated by 11 examples of flakes with both ground platforms and orthogonally ground dorsal surfaces (S4 Fig in S1 File) with edge angles <70° (Fig 11C & 11G). In addition, there is a Metal Age flake preserving the rounded edge of the axe from which it was struck on its distal end (Fig 11E).

**Fig 12:**
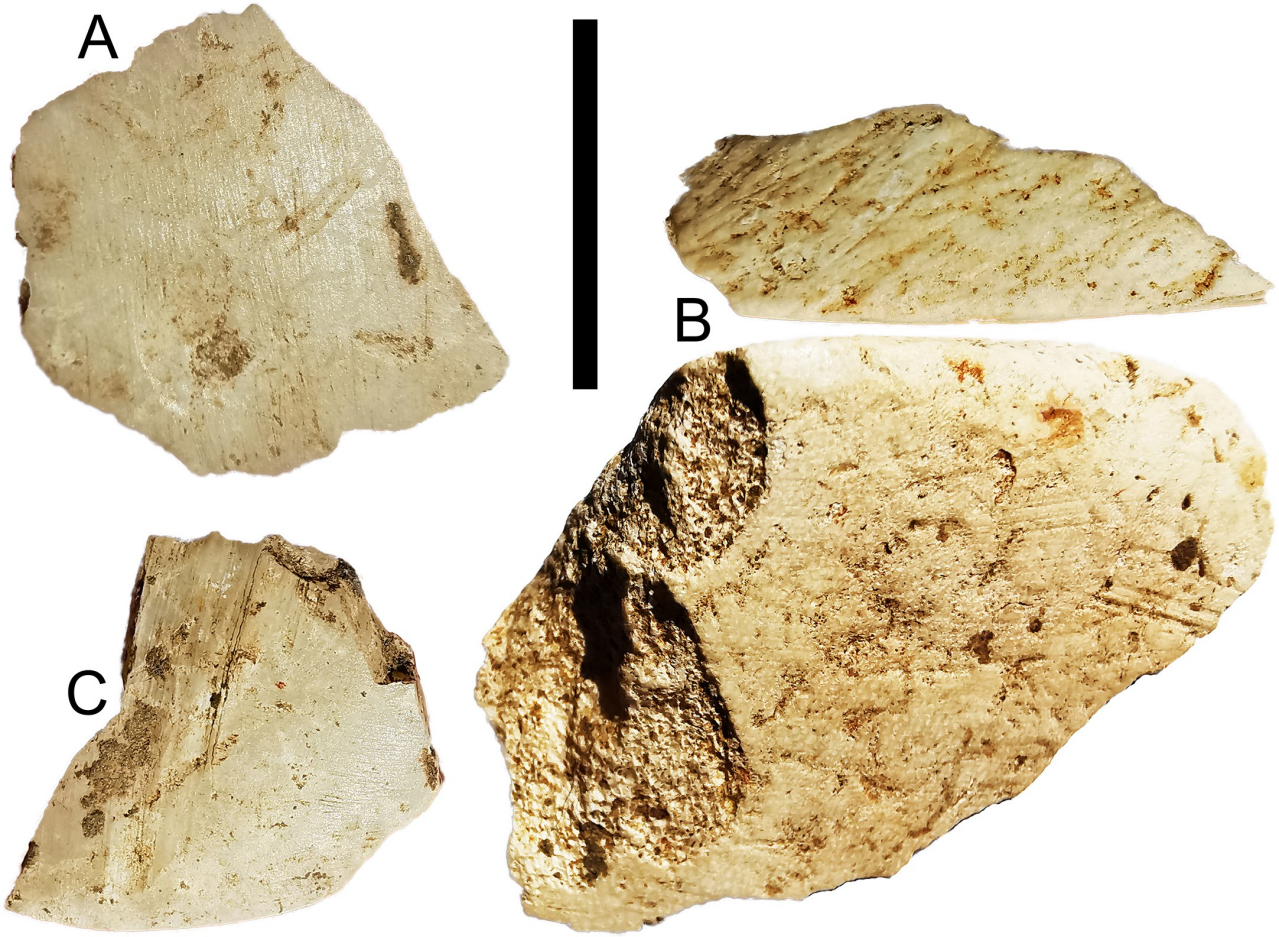
Ground shell flakes from Kelo 6. A is from layer 2a (spit 6, Metal Age), B is from layer 3 (spit 19, early Holocene), C is from layer 2b (spit 10, early Holocene). Note that both the platform and dorsal surface of B are ground. Scale bar is 1 cm long.

**Fig 13:**
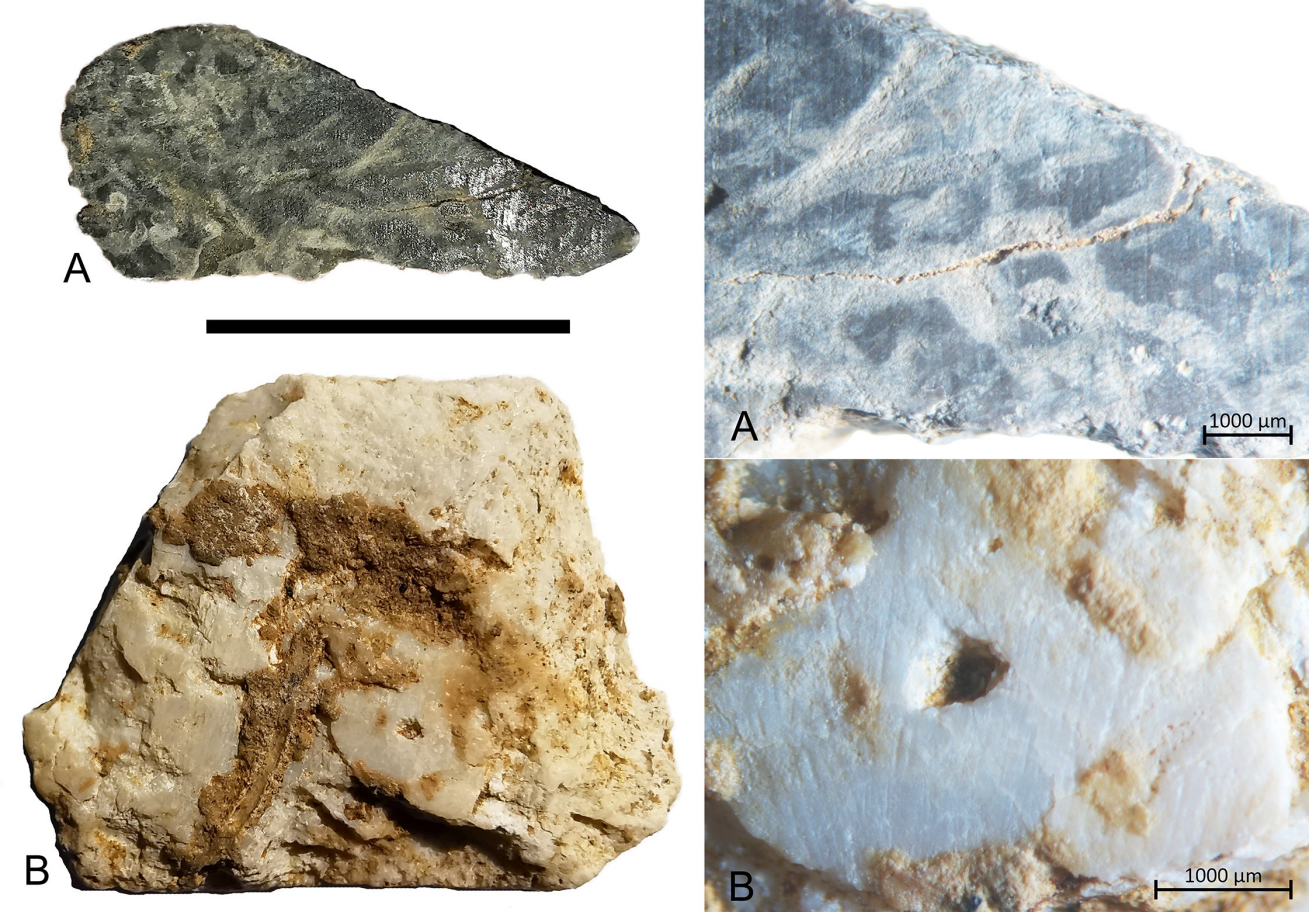
Ground shell flakes from Kelo 6 layer 4 (spit 23, terminal Pleistocene). Microscopic images shown on the right are taken with a Zeiss Stemi 2000-C microscope. Scale bar on the left is 1 cm long.

There are 23 shell flakes in the assemblage, with 4 of these having ground dorsal surfaces and 1 having both a ground platform and dorsal surface (Fig 12), suggesting much of the shell flaking is related to axe/adze production. Of the complete shell flakes, most retain some of the original shell surface (75%, N = 16), suggesting shell working entailed shorter, more expedient reduction sequences than igneous stone. The highest proportion of shell artefacts occur in the terminal Pleistocene (Table 3), including two ground shell flakes in layer 4 (Fig 13) (S2 Table in S1 File).

## Discussion

Our excavations on Obi island allow us to test human responses across the Pleistocene to Holocene transition: one of the most dramatic episodes of climate change in human history. As these are the first excavations on Obi Island the patterns identified are necessarily preliminary. Three phases of occupation are evident at the Kelo sites: an initial occupation in the terminal Pleistocene, a more intensive phase in the early Holocene, followed by a long hiatus, and lastly a Metal Age occupation in the last one thousand years.

The modelled start date for Kelo 6 layer 5 indicates occupation began by at least 17,500 years ago. Regional records show the climate became significantly drier at this time [37], which perhaps opened up Obi’s dense forests. Obi was also considerably larger than at present, being connected to neighbouring smaller islands such as Bisa and Obilatu (Fig 1B). The Kelo rockshelters are today around 2 km from the coast, but at the time of initial occupation they would have been over 6 km (Fig 1C). Sediment colour and the proportion of rocks at Kelo 6 suggest low intensity occupation in layer 5 and to some extent layer 4. In comparison to the overlying layers, the density of archaeological food remains (shell, bone, and *Celtis* sp. seeds) in layers 4 and 5 is an order of magnitude lower, but the density of lithics is comparable (Table 2). This could in part be due to preservation bias, but the shell and bone which has survived is in similar condition across phases, so the difference seems to reflect a behavioural change. We suggest the dominant lithic technological strategy in the terminal Pleistocene layers 4 and 5 was for the short-term use of small chert lithics—only 2% of chert lithics are retouched; while in the Holocene layers 1 to 3 the dominant strategy was for the long-term use of large volcanic axes–much if not all of the axe flaking is related to rejuvenating axes. Since axes are long-lived tools, they are rare and only occasionally found as whole items in archaeological deposits. The chronology of their production and use cannot rely on the recovery of complete items in stratigraphic context, but the distinctive ground facet flakes that result from their reworking are a reliable and more abundant indicator of this tool type [8].

Chert and quartz flaking technology shows little change across the sequence with bipolar, multiplatform, discoidal, and occasionally core-on-flake knapping strategies evident, and flakes of comparable size and shape being produced throughout. The dominance of chert, this range of reduction strategies, the generally small size of the lithics produced, with a mean length of 15.31 mm, as well as the occurrence of post-knapping heat damage, allies this assemblage to several penecontemporaneous and older assemblages in other parts of Wallacea [20, 38–40]. The occupants of Kelo thus brought with them a shared Wallacean technological repertoire. One of the more intensively used early Holocene chert artefacts from Kelo is the multiple notch piece (Fig 7A), with notches among the most common retouched and utilized lithics elsewhere in Wallacea [41, 42].

Cuscus are the dominant terrestrial fauna throughout the Kelo sequences, with cuscus also dominating at other Holocene sites in North Maluku including Tanjung Pinang on Morotai, Siti Nafisah on Halmahera, and Uattamadi on Kayoa (Fig 1A) [43]. Ethnographic information from Seram, immediately to the south of Obi, indicates that cuscus are the main wild terrestrial protein source, favoured for their ease of capture and their distinctive flavour [44]. Among the Nuaulu of Seram cuscus are a totemic animal in part because of their morphological similarities to humans [45]. Cuscus were an early and frequently translocated species in the broader region [e.g. 46], indicating their importance as human prey taxa. *Phalanger rothschildi* are unique to Obi however, so their occurrence from the earliest human occupation layer at Kelo suggests that they are endemic.

In layer 4 at Kelo 6 ground shell flakes appear, with the modelled centroids for the start and end of this layer 14,212 and 13,247 BP respectively. There are no igneous flakes at all in the sequence in layers 4 or 5, indicating edge-ground tool technology emerged first in shell at the end of the Pleistocene and was then manifested in igneous rock in the early Holocene. The modelled dates at Kelo 6 suggest there may have been continuity in occupation from the terminal Pleistocene into the early Holocene, with the latter phase lasting from ~13,014 to 8226 BP. There is a marked increase in occupation intensity in the early Holocene at Kelo 6, as well as the initial occupation of nearby Kelo 2. The introduction of groundstone axe technology also demonstrates technological intensification as it takes at least twice as long to grind basalt than shell, with basalt then providing functional advantages [27]. In addition to the igneous axes, the early Holocene sees the introduction of grinding stones as well as river cobble manuports that may have been used as mullers, and occasional shark vertebrae beads. Cobble manuports are also known from early to middle Holocene occupations at Tanjung Pinang on Morotai [47] and Tron Bon Lei on Alor [48, 49].

Elsewhere in Wallacea, ground shell flakes and adzes have been found from the early Holocene including Bubok on Ilin Island in the Philippines [11], Asitau Kuru on Timor [13], and most notably Golo and Buwawansi on nearby Gebe Island [12] (Fig 1A). Early Holocene shell adzes also occur at Pamwak on Manus in the Admiralty islands [50] (Fig 1A). Further instances of shell adze technology may be hidden in large assemblages of shell in which analysts are not expecting flaked and ground pieces. The Kelo finds push the date of the earliest ground shell technology in this part of the world back into the terminal Pleistocene, and they indicate this technology may have a more continuous distribution across islands than was hitherto known.

The absence of stone axe technology in the terminal Pleistocene and its subsequent appearance in the sequence suggests it was not part of the technology that enabled colonization across open-water in Wallacea and Sahul, but was independently invented in Australia and Wallacea. Elsewhere in Wallacea, including the islands both to the north and south in Maluku, edge-ground stone tools are not known until the Neolithic [47, 51]. The early Holocene groundstone axe technology at Kelo contrasts with that documented at Niah cave on Borneo: the latter is based on expedient unifacial retouch of shale cobbles [15, 52], while the former were made on volcanic stone, with bidirectional scar patterns and a lack of cortical flakes indicating they were extensively shaped through flaking before grinding. In Highland New Guinea there are isolated examples of cobbles with grinding on the cutting edge from the Last Glacial Maximum, but only in the early to middle Holocene at sites including Nombe, Kiowa, Kosipe, Kafiavana, and Manim 2, do axes occur that have been bifacially shaped then ground on their faces and sides [14, 53–58]. Duff [59: 27] and Van Heekeren [60: 165] contrast Austronesian Neolithic ground quadrangular adzes with ‘Melanesian round axes’, the latter having traits such as the cutting edge positioned mesially (i.e. an axe not an adze), an elliptical section with sides only a little less acute than the cutting edge (i.e. bifacial), and a pronounced even taper in plan from the cutting edge to the poll. This description is applicable to many recent groundstone axes from New Guinea [61, 62], with the complete Obi axes also conforming to this pattern. The Kelo sequence of axe technology for Obi Island is thus reminiscent of other Wallacean islands and Manus in its early adoption of ground shell, but mainland New Guinea in its Holocene bifacial groundstone axe industry.

Axes may have been used in arboriculture management of the forest to thin and clear competitor vegetation around desirable plants, as is practised by modern foragers in Borneo [63] as well as people on the islands to the south of Obi: Seram, Ambon, and Buru [64]. Notably, in New Guinea axes are used for hunting cuscus, where they are variously employed to fell small trees containing prey, to access dens in trees, or to improve visibility at ambush sites [65, 66]. Increased precipitation in Wallacea at the Pleistocene-Holocene transition [67] may have increased vegetation density on Obi, creating the conditions for the invention of groundstone axes. The groundstone axes of Obi may be a local manifestation of niche construction responses to rapid early Holocene environmental change, which have been documented in a variety of forms across neighbouring Sahul [68].

After ~8226 BP, there appears to have been a hiatus of several thousand years at the Kelo localities. Notably, long hiatuses beginning around this time occur elsewhere in the region, such as at Here Sorot Entapa on Kisar island [69], and Liang Lembudu on Aru island [70] (Fig 1A). At Here Sorot Entapa, where subsistence was almost exclusively maritime, abandonment may be related to sea-level rise drowning previously productive shore environments [69]. At Liang Lembudu the preceding occupation occurs when terrestrial fauna indicates a much drier savannah-like environment than exists today, with abandonment occurring at a time of loss of open habitats [70]. A similar process to Liang Lembudu may have occurred at the Kelo rockshelters, whereby further increases in forest density made the area too difficult to manage and forage in. Indeed, the flooding of the Sunda shelf ~9500 BP led to a sudden increase in precipitation in Wallacea [71] and New Guinea [37] that would have promoted forest growth. Increased precipitation may also have increased the disease load for the occupants of Obi [e.g. 72, 73–75]. However, charcoal in a lake core from western Obi spanning the last 5000 years [37], suggests abandonment of Kelo may have been a local rather than island-wide phenomenon, with occupation perhaps moving closer to the coast.

Reoccupation of the Kelo rockshelters occurs in the Metal Age in the first half of the last millennium. Despite the long hiatus, the occupation shows some remarkable similarities to the early Holocene with bifacial groundstone axe technology, the use of river cobble manuports, the predominance of cuscus hunting, and perhaps the wearing of shark vertebrae beads. The main changes in the Metal Age appear to reflect Austronesian introductions: Thin-walled pottery, pig bones, and quadrangular and occasionally polished adzes [12, 16, 18, 76]. These new technologies and subsistence practices perhaps facilitated occupation of the dense forest. Long-term technological and symbolic continuities, with the incorporation of Austronesian introductions, have also been documented on Timor and Flores in southern Wallacea [20, 38, 77].

Higher frequencies of bone, *Celtis* seeds, and lithics all suggest greater occupation intensity in the Metal Age than the early Holocene (Tables 2 and 3). On the other hand, the greater reduction intensity of chert flakes, and the greater proportion of lithics in quartz, which is probably a non-local import, suggest greater mobility in the Metal Age. The Metal Age thus presents a distinctive occupation signature to the early Holocene, with the greater intensity perhaps reflecting larger groups, and the greater mobility suggesting the shelters functioned as temporary camps rather than longer-term residences.

There is no evidence for metal production at these sites or anywhere else in prehistoric North Maluku [12], so the metal artefact from Kelo 2 may represent an exotic import. The *Oliva* shell bead tradition from Kelo is also found on the islands of Timor, Rote, and Alor in southern Wallacea [77–79], suggesting inter-island connectivity in the Metal Age. A single exotic obsidian flake from Kelo 2 also suggests long-distance connections with no known obsidian sources in North Maluku. An isolated obsidian flake from Tanjung Pinang on Morotai derives from Fergusson island off the eastern tip of New Guinea (Fig 1A), confirming long-distance connectivity; with occupation during the last 2000 years in North Maluku suggested to reflect the beginnings of the spice trade [12]. Likewise, a Metal Age long-distance exchange network featuring an exotic obsidian flake and glass beads from mainland Asia has been documented at Aru Manara on Morotai island [80].

The Kelo sites show Obi Island was occupied by at least 17,500 years ago, and they have yielded the earliest evidence for both ground shell and stone technology in Wallacea. Taking shell and igneous artefacts together, 30% of the Kelo lithic assemblage may be related to axe working, with 14% of all knapped artefacts showing traces of grinding. These are very high proportions by any standards [e.g. 53], and indicate the importance of axes to the prehistoric occupants of Obi. The investment required to produce ground axes, particularly in stone, indicates intensification on Obi at the Pleistocene-Holocene transition. In southern Wallacea, intensification in technology, as well as in body adornmentation, ritual burials, and connectivity, has been documented in the terminal Pleistocene to middle Holocene levels of Makpan, Tron Bon Lei, Here Sorot Entapa, Lene Hara, and Asitau Kuru [13, 48, 77, 81, 82]. Elsewhere in Maluku there is no record of early Holocene groundstone axes or shark vertebrae beads [12, 51], suggesting relative isolation of Obi, unlike the inter-island connectivity seen in southern Wallacea at this time [13, 83, 84]. Although the Obi groundstone axes have commonalities with some types from New Guinea, there are, as yet, no further indications of connections between the two in the early Holocene.

Small islands provide natural experiments for patterns of social change [e.g. 85], with the unique Obi record providing an independent ‘island-test’ of human innovation and intensification. There is debate as to whether intensity of human interactions or environmental change are the principal drivers of innovation in human culture [e.g. 86, 87, 88]. The Kelo excavations suggest ground shell technology emerged at a time of relatively low occupation intensity but rapid environmental change, at the end of the Pleistocene. The subsequent early Holocene innovation of bifacial groundstone axe technology is associated with a marked increase in occupation intensity, though as they appear archaeologically simultaneously it is not possible to tell if one is causally related to the other. The new excavations on Obi island suggest human innovation was a repeated response to the exceptional environmental changes of the terminal Pleistocene and early Holocene. A feedback effect may have been established whereby innovations increased occupation intensity which then engendered further innovation—in particular, deliberate niche construction.

## Supporting information

S1 File (PDF)

S1 Raw data (SAV)
