# Supplementary material for: Early ground axe technology in Wallacea: The first excavations on Obi Island
Source: PLoS One. 2020 Aug 19;15(8):e0236719. doi: 10.1371/journal.pone.0236719 (PMC7437812; doi:10.1371/journal.pone.0236719)
Supplement: S1 File — (PDF) [file pone.0236719.s001.pdf]

# - Supporting Information -

Early ground axe technology in Wallacea: The first excavations on Obi Island

This is a supplementary file containing information on the excavation of the stratigraphic contexts differentiated at Kelo 6 (S1), results of the dating Bayesian model (S2), methods, results, and interpretation of the modelled depositional discontinuity (S3), a table showing shell, igneous, and ground flakes by layer and spit (S4), and two figures showing the relationship of different types of axe flake to the whole axe and a redirecting igneous flake preserving a bifacial platform from the surface of Kelo 6 (S5).

Supporting Information 1

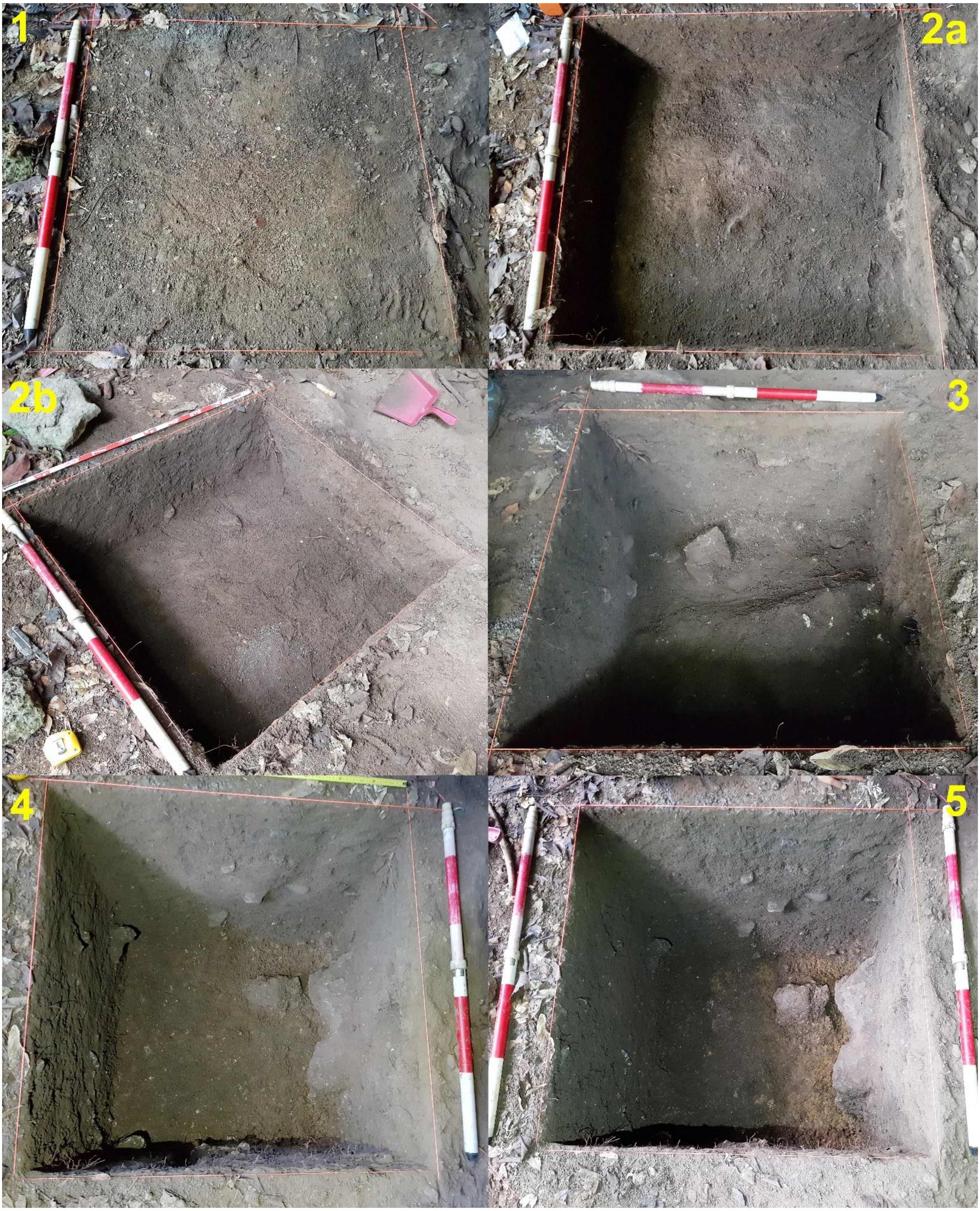

Figure S1: The upper surfaces of the six stratigraphic contexts differentiated during the Kelo 6 excavation. The scale bar is on the side of the excavation furthest from the shelter wall for contexts 1, 2a, and 2b, and the side closest to the shelter wall for contexts 3, 4, and 5.

Supporting Information 2

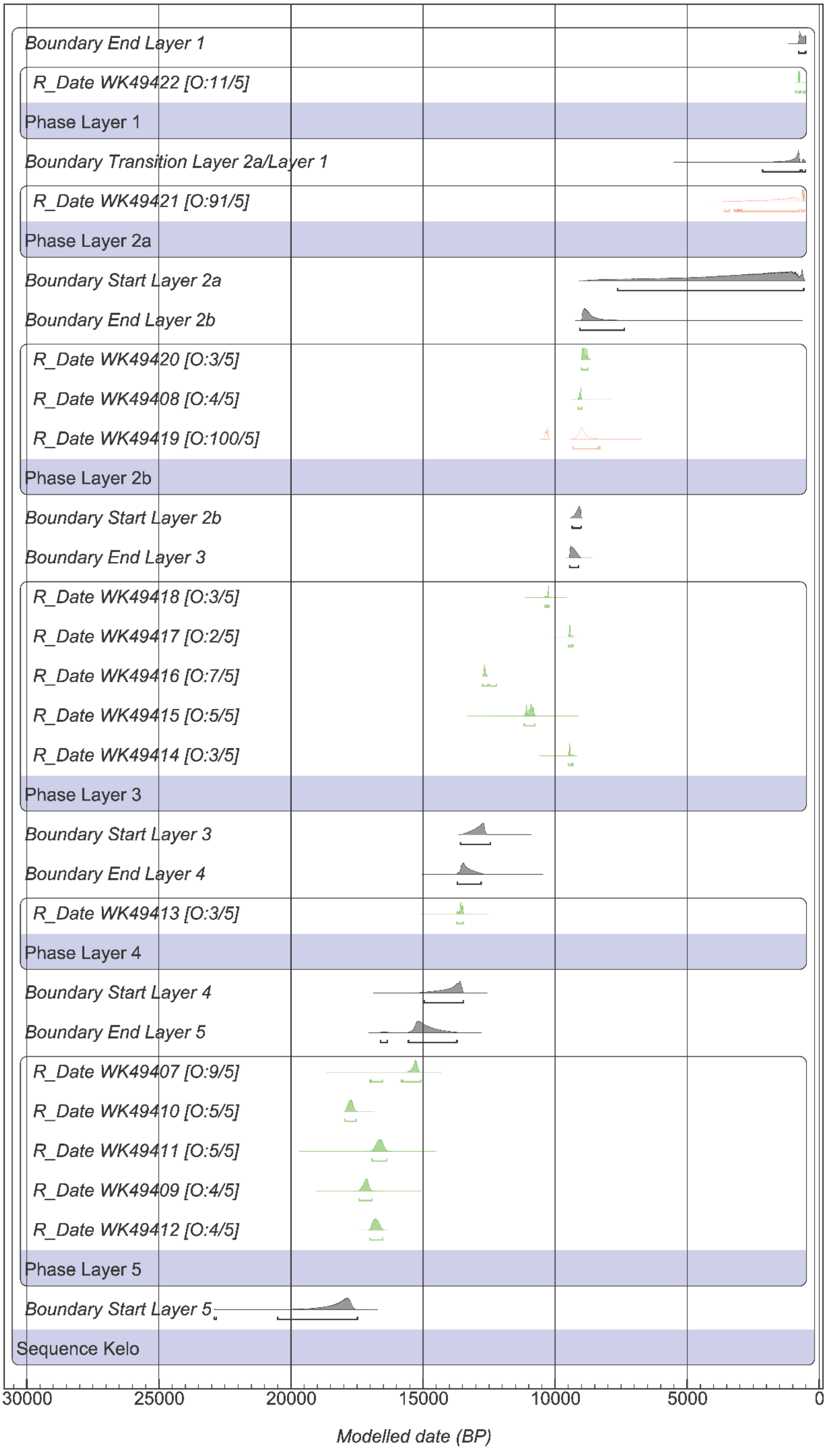

Figure S2: Bayesian model of the radiocarbon dates from Kelo 6. Calibrations were made using OxCal v4.3.2 (Ramsey 2017), and IntCal13 for charcoal and aragonite seeds or Marine13 for marine shell (Reimer et al. 2013). Pale probability distributions represent the calibrated, unmodelled date, whilst filled distributions represent the modelled date. Green indicates dates which fit well within the model while red indicates outliers. Modelled start and end distributions for the different layers are in grey. The brackets beneath the distributions represent the 95.4% probability range. Prior and posterior outlier probabilities are given in brackets following the sample name in the form [O: posterior/prior].

Supporting Information 3

The *Difference* function in OxCal was used to examine whether the apparent gap in the radiocarbon record for Kelo 6 between layers 2b and 2a did indeed represent a 'true' inconsistency in the record. This function subtracts one probability distribution function from another. If zero is included in the 95.4% probability range, the probability distribution functions are regarded as indistinguishable at 95.4%. Thus, the modelled distribution for the start of layer 2a was subtracted from the modelled distribution of the end of layer 2b.

Table S1: The results of the *Difference* function. Note that the difference range does not include 0.

NAME	MODELLED (BP)	DIFFERENCE (YRS)		
		from	to	%
**DIFFERENCE 2B/2A**		749	8,288.5	95.4
**PRIOR END_LAYER_2B**	9,068 – 7,374			
**PRIOR START_LAYER_2A**	7,635 - 586			

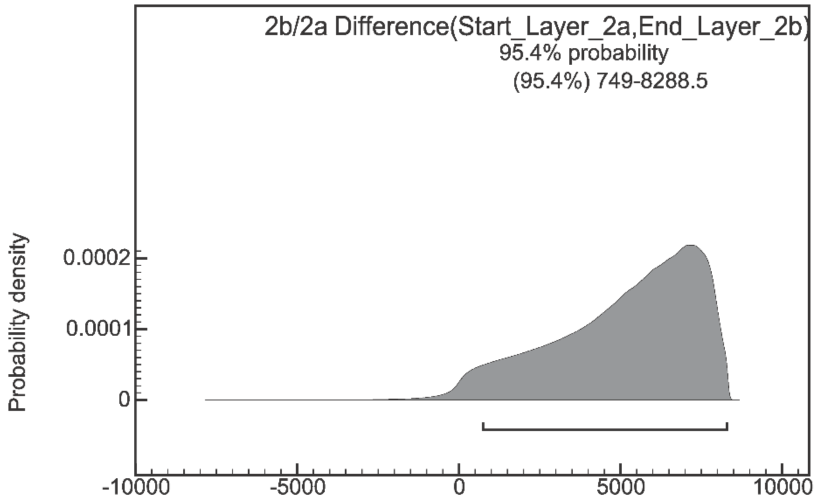

Figure S3: The Difference *(yrs) between the modelled end of layer 2b and the start of layer 2a at Kelo 6. The bracket beneath the distribution represent the 95.4% probability range.*

As the mean age of each modelled boundary falls outside the 95.4% CI of the other, and the 95.4% CI of the difference model does not cross the 0 interval, we can state that there is a 95.4% probability of a discontinuity in the record between layers 2b and 2a.

Supporting Information 4

Table S2: Breakdown of the distribution of shell, igneous, and axe rejuventation flakes (those with ground facets) by phase, layer, and spit for Kelo2 and Kelo 6. Axe rejuvenation flakes were exclusively on shell and igneous material.

Site	Phase	Layer	Spit	Shell	Igneous	Axe rejuvenation
Kelo 2	Metal Age	1	1	0	3	1
Kelo 2	Metal Age	2	2	0	6	1
Kelo 2	Metal Age	2	3	0	7	3
Kelo 2	Metal Age	2	4	0	6	4
Kelo 2	Early Holocene	3	5	0	2	2
Kelo 6	Metal Age	1	1	1	0	0
Kelo 6	Metal Age	1	2	2	9	4
Kelo 6	Metal Age	1	3	2	13	3
Kelo 6	Metal Age	1	4	0	16	10
Kelo 6	Metal Age	2a	5	0	10	3
Kelo 6	Metal Age	2a	6	1	16	9
Kelo 6	Metal Age	2a	7	1	14	9
Kelo 6	Early Holocene	2b	8	1	5	4
Kelo 6	Early Holocene	2b	9	0	4	3
Kelo 6	Early Holocene	2b	10	1	7	5
Kelo 6	Early Holocene	2b	11	0	3	1
Kelo 6	Early Holocene	2b	12	0	5	4
Kelo 6	Early Holocene	3	13	0	3	3
Kelo 6	Early Holocene	3	14	0	1	1
Kelo 6	Early Holocene	3	15	0	1	0
Kelo 6	Early Holocene	3	16	0	3	1
Kelo 6	Early Holocene	3	17	0	5	1
Kelo 6	Early Holocene	3	18	0	0	0
Kelo 6	Early Holocene	3	19	2	2	2
Kelo 6	Early Holocene	3	20	0	4	1
Kelo 6	Terminal Pleistocene	4	21	2	0	0
Kelo 6	Terminal Pleistocene	4	22	3	0	0
Kelo 6	Terminal Pleistocene	4	23	3	0	2
Kelo 6	Terminal Pleistocene	5	24	1	0	0
Kelo 6	Terminal Pleistocene	5	25	0	0	0
Kelo 6	Terminal Pleistocene	5	26	3	0	0

Supporting Information 5

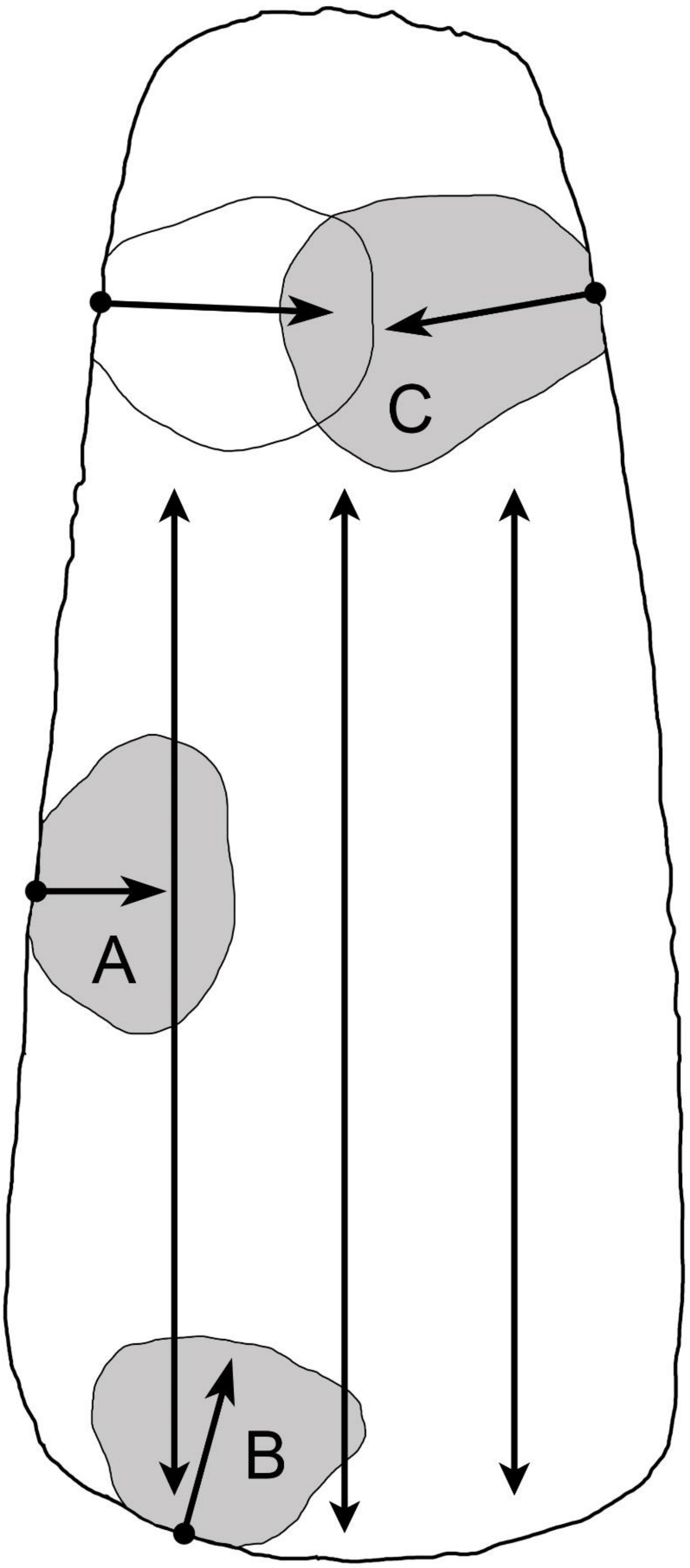

Figure S4. The relationship of different types of axe flakes to a whole axe. Double-ended arrows show the main direction of grinding striations, arrows from dots show flake percussion directions. For flake A, removed from the side of the axe, the percussion axis is orthogonal to the axis of the striations. For flake B, removed from the cutting edge of the axe, the percussion axis is sub-parallel to the striations. For flake C the flake scar pattern is bidirectional due to the invasive flaking to shape the axe from either side.

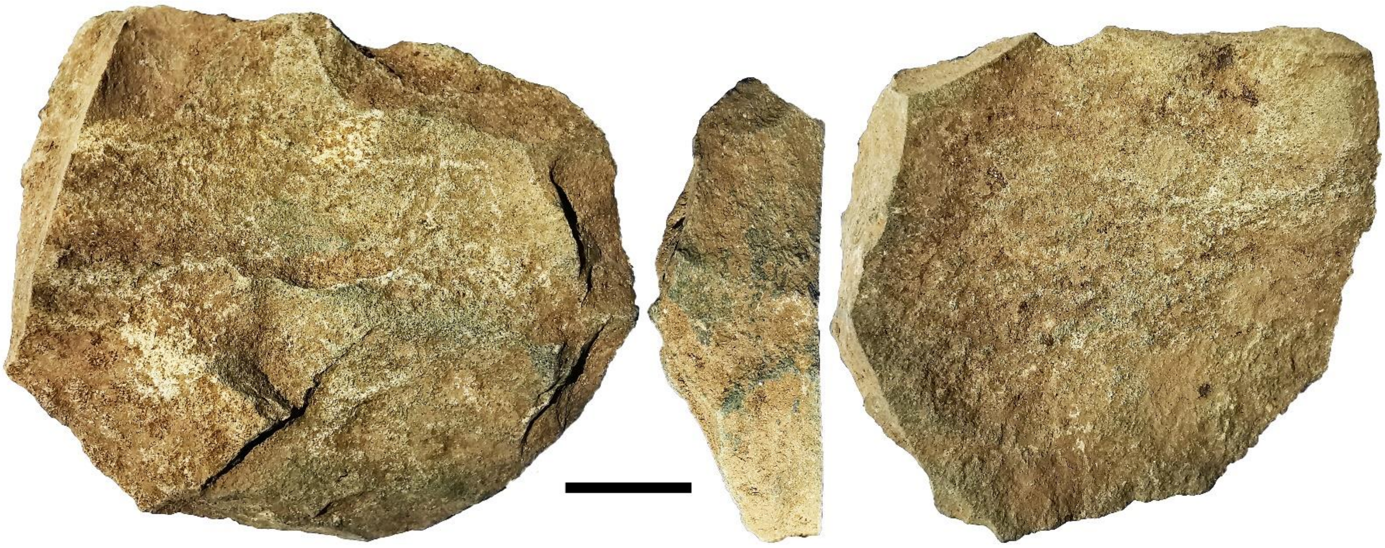

Figure S5. Redirecting igneous flake from the surface of Kelo 6 with a bidirectional platform preserved on the lateral edge.
